# Long Range Coherent Energy Transfer in Artificial Multichromophoric Antenna Systems—A Case of Breaking Kasha's Rule

**DOI:** 10.1002/anie.202513001

**Published:** 2025-08-01

**Authors:** Maryam Nazari Haghighi Pashaki, Caroline D. Bösch, Florian Garo, Ambre Blanc, Marco Marazzi, Ariana Rondi, Michela Gazzetto, Maryam Akbarimoosavi, jean‐christophe Tremblay, Simon Matthias Langenegger, Antonio Monari, Robert Häner, Thomas Feurer, Andrea Cannizzo

**Affiliations:** ^1^ Institute of Applied Physics University of Bern Sidlerstrasse 5 Bern CH‐3012 Switzerland; ^2^ Department of Chemistry, Biochemistry and Pharmaceutical Sciences University of Bern Freiestrasse 3 Bern CH‐3012 Switzerland; ^3^ Université de Lorraine & CNRS LPCT UMR 7019 F‐5400 Nancy, Metz France; ^4^ Departamento de Química Analítica Química Física e Ingeniería Química, Functional Molecular Systems (FuMSys) group, Universidad de Alcalá Ctra. Madrid‐Barcelona Km. 33 Alcalá de Henares (Madrid) 600 E‐28805 Spain; ^5^ Universidad de Alcalá Instituto de Investigación Química “Andrés M. del Río” (IQAR) Ctra. Madrid‐Barcelona Km. 33 Alcalá de Henares (Madrid) 600 E‐28805 Spain; ^6^ Université Paris Cité and CNRS ITODYS, Paris F‐75006 France

**Keywords:** Anti‐Kasha photochemistry, Coherent energy transfer, DNA‐guided design, Multichromophoric complex, Phenanthrene

## Abstract

Ultrafast long‐range energy transfer with unitary quantum‐yield has been studied in a novel family of artificial DNA‐guided multichromophoric antenna complexes with phenanthrene moieties as light harvesters. By means of femtosecond transient absorption spectroscopy and time‐dependent density function theory calculations, we demonstrate that the highly efficient long‐range energy transfer occurs via a coherent mechanism. It is enabled by photoexcitation of higher lying delocalized excited states of phenanthrene, and hence, can be labelled anti‐Kasha in nature. Moreover, we show the important contribution of exciplex formation between acceptor and closest donor.

## Graphical abstract

We investigated a unitary quantum‐yield long‐range energy transfer in a novel family of artificial DNA‐guided multichromophoric antenna complexes. We demonstrated that it occurs via a coherent mechanism enabled by the photoexcitation of delocalized higher excited states of the complex, being in this respect an anti‐Kasha process. The importance of exciplex formation between acceptor and closest donor was also studied.

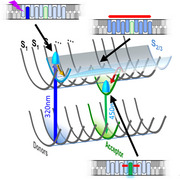

## Introduction

In the last years, we have explored the photophysical properties of multichromophoric systems (MCSs) containing phenanthrene monomers as donors to design light harvesting systems in a variety of self‐assembling polymeric structures.^[^ ^1^, ^2^, ^3^ ^]^ These assemblies exhibit an efficient energy transfer (ET) over tens to hundreds of nanometers with unitary quantum yield (QY) in aqueous solutions at room temperature. Additionally, their broadband absorption is extremely intense (molar extinction coefficients >10^6^ M^−1^cm^−1^). These properties make them suitable candidates to trigger, within a few nanoseconds and even stimulated by solar light irradiation, catalytic processes involving multiple excitations such as water splitting, which requires four photoexcited electrons (see Supporting Information for a comparison of excitation rates in MCS). Another essential property provided by such MCSs is the directional energy transfer over several nanometers, which is a necessary prerequisite for efficiently directing the excitation energy toward a specific reaction center. This aspect should not be underestimated since the transfer length influences the number of excitations per reaction center achievable within the time window of the photocatalytic reaction and, thus, the optimal maximal extension of the MCS.

Although the strong interchromophoric electronic coupling between excitonic states is indicative of an efficient energy transfer, the nature of the ET process (quantum coherent or fast hopping^[^ ^4^, ^5^, ^6^ ^]^), the involved electronic states, and the nuclear dynamics that might assist the ET are still debated. In addition, we were intrigued by the potential specific role of phenanthrene, since such an exceptionally efficient ET was observed only in MCSs using phenanthrenes as donor chromophores.

To address these questions and, on top of that, to study dynamics of the long‐range ET in MCSs, we focused on DNA‐guided antenna systems. DNA provides a versatile and reliable supramolecular scaffold for the controlled assembly of multichromophoric arrays.^[^ ^7^, ^8^, ^9^, ^10^, ^11^, ^12^ ^]^ Complementary DNA strands are used to incorporate dyes in a well‐defined conformation, thus enabling the arrangement of aromatic chromophores in a π‐tacked configuration.^[^ ^13^, ^14^, ^15^, ^16^ ^]^ Our perspective here is to accelerate the transition from the trial and error phase to a rational molecular design approach.^[^ ^17^, ^18^, ^19^ ^]^

In this study we adopted fs time‐resolved electronic spectroscopy and quantum chemical molecular modelling to unravel the ET process in real‐time. We carried out a comparative study of the monomer light‐harvester (the energy donor), a stack of several light‐harvesters only and a stack where one light‐harvester is replaced by an energy acceptor, namely a pyrene. This strategy allowed us to characterize intramolecular processes and spectral signatures of individual donors, before investigating the new processes and electronic states generated by the stacking of several donors, and eventually identifying the processes that are enabled by the presence of the pyrene acceptor.

The study on the photophysics of the phenanthrene monomer was previously reported^[^ ^20^ ^]^ identifying a complex photocycle originating from population branching and dynamics through a conical intersection (CI) between the lowest, *S_A_*, and a higher singlet excited‐state, *S_B_*. The lowest lying bright transition from the ground state (*S_0_*) populates *S_B_* and the excited population relaxes toward *S_A_* through the CI. This occurs first via an ultrafast internal conversion (IC) involving ∼80% of the excited population and then via a thermally activated IC of the remaining 20% of the population in ∼600 fs. Despite *S_B_* being the absorption bright state, the emission stems mainly from *S_A_*. These results constitute the starting point for the present study where we compare phenanthrene monomers with an array of 10 stacked phenanthrene units with and without pyrene as energy acceptor.

**Figure 1:**
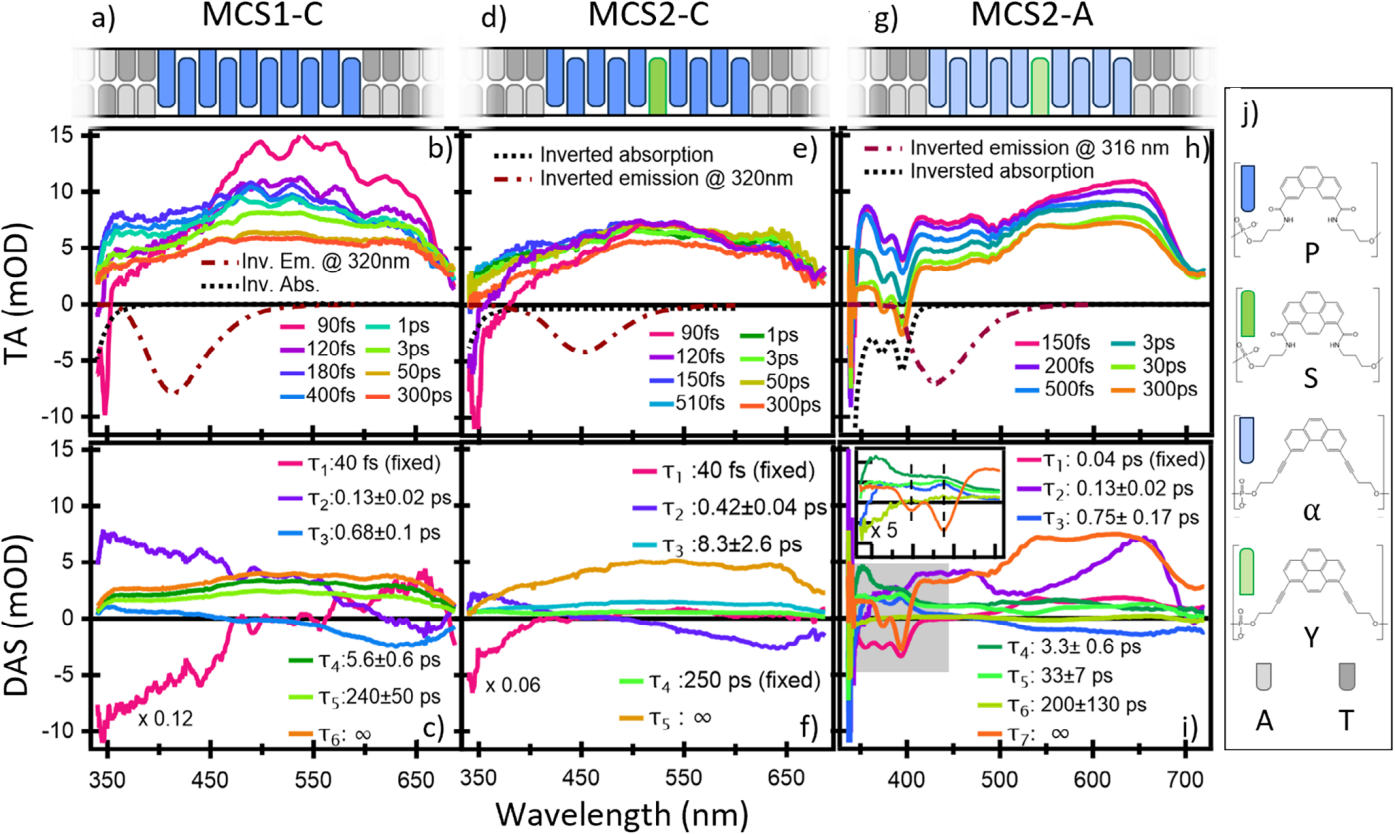
(Top) Investigated multichromophoric systems (MCSs). Each MCS is a duplex of two single strands containing adenine A), thymine (T), carboxamide‐linked phenanthrene (P) and pyrene (S) and alkynyl‐linked analogues (**α **and **Y**, respectively). For the sake of clarity, the case of the MCS1‐A is not shown (see Supporting Information). See panel (j) and Scheme S1 in Supporting Information for the molecular structures. (2nd row) Selection of ultrafast transient absorption spectra excited at 320 nm. Inverted steady state absorption and emission spectra are also shown (see Figure S1). (3rd row) Time‐Spectrum decomposition analysis (details in Supporting Information): decay associated spectra (DAS) and relative decay time constants are shown. The inset in the panel (i) shows the DAS components related to Förster resonance ET (FRET; see text for more details) and the long‐lived one in the spectral range around the pyrene ground state bleaching (GSB).

In addition, we compared two MCSs with the same structure but with two different linkers, namely carboxamide and alkynyl moieties (Scheme S1), to address i) their effect on the ET process and ii) the role of the acceptor‐closest‐donor (AcD) exciplex formation on the efficiency of the ET. Indeed, the functionalization, necessary to incorporate non‐nucleotide units in the DNA backbone, can considerably affect the interchromophore coupling and the optical properties of the MCSs. The reason to choose these two linkers is that the former allows an exciplex formation between AcD^[^ ^1^ ^]^ while the latter induces a weaker AcD coupling, hence preventing exciplex formation (§SI.3). The investigated MCSs are listed in Table S1, where the notation is also explained (see Scheme S1 in Supporting Information for the molecular structures). The ones relevant to the discussion are also sketched in the top panel of Figure 1.

Our comprehensive study proves that the efficient ET QY relies on an interplay between the short‐lived, high‐lying delocalized bright states and the long‐lived, low‐lying dark states, which exhibit a strong localized character and are connected by conical intersections. The coherent ET occurs within the delocalized states, making it, to the best of our knowledge, the first reported example of a fully anti‐Kasha coherent ET process. Indeed, other reported anti‐Kasha transfer processes in artificial systems involve “classical” (incoherent), short‐range processes in dyads or triads, and rely on an unusually long higher excited state lifetime or a fast conformational relaxation^[^ ^19^ ^]^ What we observe here is very different: a fully electronic process that involves the formation of delocalized excitonic states able to transfer instantaneously energies over several nanometers. In this regard, the present systems represent a rare successful attempt to mimic natural light harvesting systems. Note that we use the term anti‐Kasha in more general terms relating to any photophysicochemical process that depends on the nature of the photoexcited state.^[^ ^19^ ^]^ Moreover, our studies prove that the astonishingly long‐range coherent ET processes within higher excited states of π‐stacked systems can occur efficiently at ambient temperatures. This prevents losses during the conversion of the harvested photon energy and indicates a realistic route toward large and efficient visible light harvesting systems based on π‐stacked MCSs.

## Results and Discussion

For sake of readability, MCSs without and with the acceptor are labelled “1” and “2”, while the suffixes –C and –A refer to MCSs with carboxamide and alkynyl linkers, respectively. When the labels or the suffixes are not specified (e.g., MCS‐C or MCS2) we refer to both the respective MCSs (e.g., MCS‐C stands for MCS1‐C and MCS2‐C, whereas MCS2 for MCS2‐C and MCS2‐A).

A detailed study of the steady state absorption and emission from the different samples is reported in refs. [2, 21, 22, 23] and summarized in the Supporting Information. MCS1 show the characteristic phenanthrene emission at 415 nm upon excitation at 316–320 nm, i.e., at the maximum of the absorption band, which is found to be completely quenched by insertion of an acceptor in MCS2 (Figure S1). This is accompanied by the appearance of an emission at 450 nm in MCS2‐C and 430 nm in MCS2‐A, upon excitation of the phenanthrene absorption band. Indeed, the fraction of directly excited acceptors at 320 nm is negligible (Figure S2 and relative discussion). The quenching mechanism was found to be a very efficient ET from the π‐stacked phenanthrenes to the pyrene, which is independent of the distance between the photoexcited donor and the acceptor, at least over several tens of chromophores. Relevant for the following discussion, it was reported^[^ ^1^ ^]^ that in MCS2‐C the ET leads to the formation of an exciplex between the carboxamide‐linked pyrene (**S**) and the closest carboxamide‐linked phenanthrene (**P**). Conversely, in MCS2‐A, the alkynyl‐linked pyrene (**Y**) and phenanthrene (**α**) do not form any exciplex and the emission stems from **Y** only.

The ultrafast transient absorption (TA) spectroscopy results (for detailed information on the setup and sample handling we refer to Supporting Information and literature.^[^ ^20^, ^24^ ^]^) are summarized in Figure 1, where a selection of TA spectra and the spectro‐temporal decomposition analysis for each sample are shown. Table 1 reports the time constants from the analysis, grouped according to the relaxation process to which they are assigned, and, for comparison, those for the monomer P (from ref [20]).

**Table 1:** Time constants from the analysis in Figure 1 and [20], grouped according to the relaxation process they mainly account for. Notation: FC, Frank–Condon; IC, internal conversion; Expl, exciplex formation; VET, vibrational energy transfer and cooling; CR, conformational relaxation; and FL fluorescence.

	$\tau_{IC}^{\prime }$	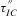	$\tau_{IC}^{Expl}$	τ_*VET*_	τ_*CR*_
MCS1‐C	0.13 ± 0.02	0.68 ± 0.10	–	5.6 ± 0.6	240 ± 50
MCS2‐C			0.42 ± 0.04	8.3 ± 2.6	250^(2)^
MCS1‐A	0.24 ± 0.03.		–	4.0 ± 0.5	27 ± 9
MCS2‐A	0.13 ± 0.02	0.75 ± 0.17		3.3 ± 0.6	33 ± 7; 200 ± 130
P^(^ ^*^ ^)^	0.10 ± 0.03	0.60 ± 0.06	–	12.1 ± 1.7	300 ± 60
	τ_*FC*_ is pulse limited^(1)^ and τ_*FL*_ = ∞^(3)^ for all samples				

(*) from Ref. [20] (see also Figure S4); ^(1)^ fixed to the instrumental response (40 fs);^(2)^ fixed; ^(3)^ set to ∞ being longer than the spanned range (0–400 ps).

We first comment on MCS‐C (panels (a) to (f)) starting from MCS1‐C, whose spectro‐temporal response is similar to that of the **P** monomer (Figure S4, Table 1), but with few significant differences. The first component τ_1_ describes a strong negative signal at *λ* < 450 nm centered at *λ* < 350 nm, resembling neither the inverted steady state optical absorption (OA) nor the emission band. Instead, it is very similar to the first decay associated spectrum (DAS) of the monomer **P** (Figure S4), which describes a stimulated emission (SE) decaying within the same time scale, and which is due to the departure from the Frank–Condon (FC) region of *S_B_*.^[^ ^20^ ^]^ This observation, plus its spectral position being closer to the OA band (inverted dashed spectrum in Figure 1b) than the steady state emission, allows us to assign it to a pulse‐limited (<40 fs) SE from the FC region of the bright state, *S_B_*. The two decays $\tau_{2}∼130$ fs and $\tau_{3}∼680$ fs are biphasic kinetics describing the decay of a positive signal, i.e., an excited state absorption (ESA) at *λ* < 550 nm, and a rise at longer wavelengths, which is assigned to the dynamics of the two higher transient states (*S_A_* and *S_B_*). The temporal evolution of these signals reflects both the *S_B_* relaxation and the internal conversion (IC) of equilibrated *S_B_* state toward *S_A_*. Remarkably, the main differences with respect to the **P** monomers are observed in these two signals, which are now broader and more unstructured spectra (see Figure S5 and Figure S6 for a direct comparison), indicating an enhanced delocalized character of *S_B_*. The fourth DAS describes a signal decay on a time scale typical of vibrational energy transfer (VET). This was observed also in the **P** monomer and rationalized as depopulation from the hot *S_A._*.^[^ ^20^ ^]^ In addition, the shape of this DAS and those of the two DASs with longer time constants strongly resemble the corresponding monomer's features (Figure S5), suggesting that *S_A_*, in contrast to *S_B_*, maintains a localized character. The τ_5_ component could be hastily assigned to rotational diffusion since it occurs in **P** monomers within 300 ps. Yet, MCSs should have a much slower rotational diffusion time constant than a **P** monomer, because of its size and shape. Furthermore, even though this DAS describes a change in the spectral shape at *λ*>550 nm, we can exclude any population dynamics, because the QY of the emission (QY_Em_) is the same (∼ 0.2) for both the **P** monomers as well as MCS1‐C. Thus, we assign the τ_5_ component to a modulation of electronic properties (such as optical cross‐sections) caused by a slow configurational relaxation (CR) of the entire chain. A similar behavior was found in the **P** monomer photocycle,^[^ ^20^ ^]^ supporting its presence in MCS1‐C and suggesting that it relates to a structural relaxation of the single chromophore, but hindered by the stacking. The sixth component accounts for the depopulation of the equilibrated *S_A_* and equates the fluorescence (FL) lifetime. Following these assignments and the consistency with the monomer behavior, we rename the time constants in Figure 1c from τ_1_ to τ_6_ as τ_*FC*_, τ′_*IC*_, τ′′_*IC*_, τ_*VET*_, τ_*CR*_ and τ_*FL*_, as reported in Table 1. Although this assignment suggests the presence of delocalized *S_B_* states in the stacked **P**s, which are likely responsible for the long‐range ET (see also the discussion on MCS2‐C), the low‐lying *S_A_* states maintain a localized character. This photocycle, inferred only from the experimental results, is fully supported by the quantum chemical calculations discussed at the end of this section.

**Figure 2:**
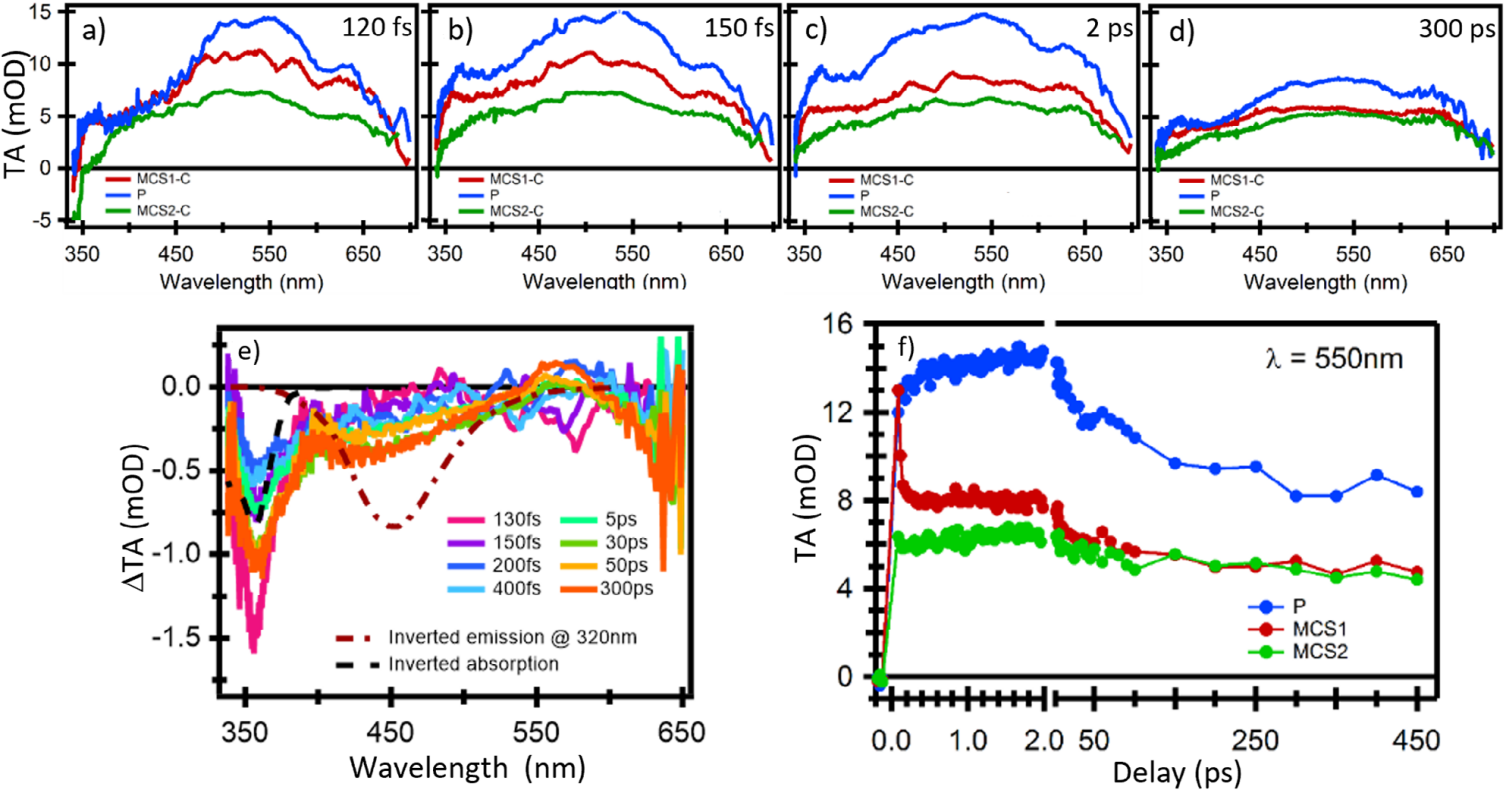
a)–d) Comparison of TA spectra from **P** monomer, MCS1‐C and MCS2‐C at four representative time delays (from Ref [20], Figure 1b and e, respectively). e) Differential spectra of MCS2‐C minus MCS1‐C, with MCS1 normalized to maximize the overlap with the MCS2‐C signal at *λ* > 550 nm (see also Figure S7). Inverted S steady state absorption and excimer emission spectra excited at 320 nm are also shown. f) Comparison of kinetic traces from phenanthrene monomer, MCS1 and MCS2 at the signal maximum (550 nm).

To identify the dynamics of the donor‐to‐acceptor ET processes and the role of higher delocalized states on the ET, we inserted an acceptor **S** in the **P** stack (MCS2‐C). We chose to place it in the middle of the stack to minimize as much as possible interactions with the DNA scaffold and to obtain more significant information on the AcD coupling (Figure 1d–f). The overall picture is qualitatively similar to the one for MCS1‐C, but important differences in intensity and spectral shape of some dynamics can be identified. These dissimilarities are well highlighted by comparing the **P** monomer, the MCS1‐C and the MCS2‐C TA signals at few selected times, as shown in Figure 2a–d. This comparison reveals that the MCS2‐C exhibits almost no dynamics and its final state is populated immediately. Conversely, MCS1‐C is initially close to a monomer signal and afterward converges asymptotically to a signal close to the one from the long‐lived state of MCS2‐C. The nature of this relaxed state is known from the steady state measurements (see Figure S1 and relative discussion) and it is assigned to the harvested energy localized on the pyrene‐closest‐phenanthrene (**S**c**P**) exciplex. This strong similarity and the bell‐shape of the MCS1‐C steady state emission hint at a long‐lived state of MCS1‐C with a localized character of the *S_A_* state on one of the **P** units. The fact that the TA signals from the relaxed MCS‐C show common features suggests that most of the exciplex TA signal originates from an electronic density localized on the **P** moiety of the **S**c**P** exciplex.

The main difference is observed at <500 nm, where the MCS2‐C signal is systematically smaller than that of MCS1‐C. To clarify its origin, differential spectra are shown in Figure 2e where we identify two negative bands centered at 360 and 450 nm. They resemble in position and shape the steady states **S** absorption and the exciplex emission (inverted dashed spectra in Figure 1b and Figure S1), respectively. Hence, the former must be ground state bleaching (GSB) from excited **S** and the latter SE from the exciplex. The presence of the fully developed GSB signal in the very first TA spectrum definitively reveals that the pyrene excitation is instantaneous, at least within our time resolution. Remarkably the exciplex emission at 450 nm, is delayed with respect to time zero and the pyrene excitation by approximately 300 fs (see Figure S7B). These results, together with the lack of any spectral dynamics, allow us to conclude that the energy is localized on the **S**c**P** pair immediately after excitation.

Regarding the evolution of MCS1‐C with respect to **P** monomer and MCS2‐C, a visual inspection of Figure 2a–d suggests that MCS1‐C is initially closer to a monomer‐like behavior (see also time‐zero spectra in Figure S6A) and eventually converges, within the lifetime of the delocalized states (*S_B_*), to a spectrum much closer to MCS2‐C. This is corroborated by the kinetic trace at the signal maximum at 550 nm (Figure 2f). Moreover, as also seen in Figure 2f, the insertion of the acceptor in MCS2‐C quenches the entire population in the delocalized state, making the ET process site‐specific.

Concerning the assignment of the spectral components, we cannot adopt the same explanation and analysis used for MCS1‐C, since the emissive species is not a **P** but an exciplex and the energy is localized on the **S**c**P** pair just after pulse excitation. Yet, we may rationalize the analysis for MCS2‐C in view of the aforementioned considerations: the first component describes a pulse‐limited signal and is due to the departure of the excited **P** from the FC region and the population of the **S**c**P** pair. According to previous studies reporting exciplex formation^[^ ^1^ ^]^ and to the discussion on Figure 2e, the second *DAS* must describe the formation of the exciplex and its emissive state, instead of the depopulation of the delocalized states. The 8.3 and the 250 ps can be rationalized analogously as τ_*VET*_ and τ_*CR*_ for MCS1‐C, respectively, since in both systems the former describes the vibrational energy dissipation and the latter the slow conformational relaxation triggered by the energy localization on one section of MCS.

**Figure 3:**
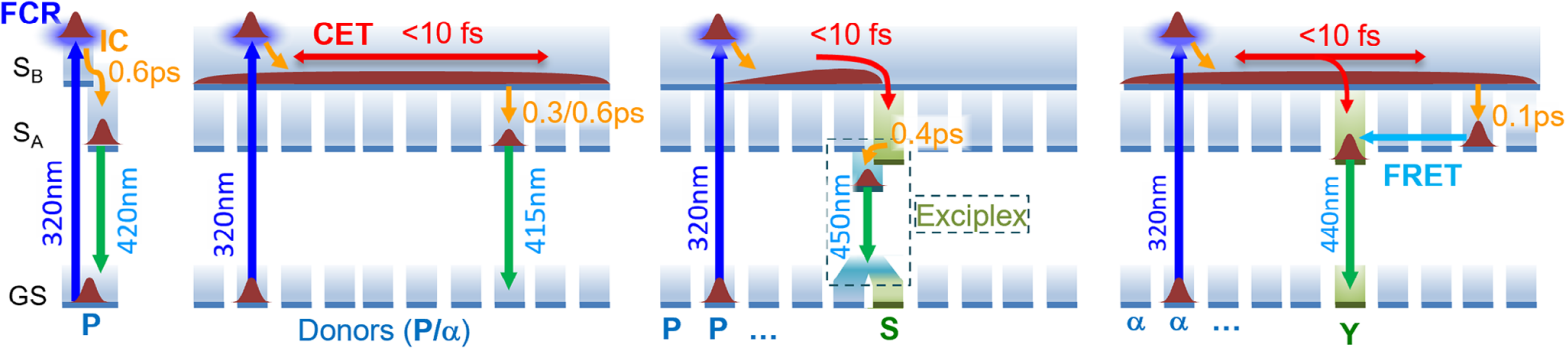
Proposed energy transfer (ET) mechanisms. The MCS1's photocycle is the same regardless the linkers: the MCS is initially excited to a higher excited state localized on one of the phenanthrene (**P** or **α**); then the excitation relaxes in few tens of fs, or less, from the Frank–Condon region (**FCR**) to the delocalized higher excited states (***S_B_***) and is spread over the whole column. Eventually it localizes in 680/240 fs (MCS1‐C and ‐A, respectively) on one of the lowest phenanthrene‐centered excited states (***S_A_***), originating the 415 nm emission. In the MCS2‐C the acceptor is so strongly coupled with *S_B_*, due to the **ScP** exciplex formation, to let all the photon‐energy to be immediately transferred to the pyrene. Conversely the weaker, but still sizable, coupling in the MCS2‐A allows for a *S_B_* lifetime of ≈ 100fs and part of the energy can populate randomly one of the *S_A_* states, which eventually transfer their excitation via **FRET**. For clarity, we omitted the processes nonrelevant to the ET, i.e., branching at the conical intersection, cooling, and conformational relaxation. Notation: IC, internal conversion; **CET**, coherent ET; and **GS**, ground state. For completeness, a simplified photocycle of the phenanthrene monomer is shown (see Ref [20] and Supporting Information for a more complete scheme). In this case, *S_B_* refers trivially to higher excited states of the monomer.

The general picture derived from this comparative study of **P** monomers, MCS1‐C and MCS2‐C, is the following: just after photoexcitation, MCS‐C is promoted to higher levels of *S_B_*, which are weakly coupled to the corresponding states of the nearest neighbor chromophores, and hence, cannot initially form delocalized states. This is supported by the similarity of the OA bands of the three systems. Upon population of the relaxed *S_B_* state, the systems undergo a conformational change, which accompanies the formation of the delocalized states that ultimately are responsible for the long‐range and coherent ET. This process surely increases the interchromophore interaction, hence the conformational change is very likely related to an increase in packing. In MCS1‐C the energy resides in *S_B_* states, within the same time scale observed in **P** monomers, before it populates *S_A_* (**P** and MCS1 in Figure 3). This process is accompanied by energy localization on one of the stacked **P**s. In MCS2‐C, the energy is localized immediately (within a few tens of fs or less) on the **S**c**P** pair (MCS2‐C in Figure 3). Since the characteristic rise time of the exciplex emission and the depopulation of the *S_B_* state in MCS1‐C are very similar, we propose the following model for the exciplex photocycle: initially the energy is localized on the *S_B_* state of the **P** closest to the acceptor, which is slightly distorted (likely lower in energy) by the presence of **S**. This state is still strongly coupled with the delocalized states on the other **P**s but the distortion makes it acting as an efficient energy trap. The formation of the exciplex emission corresponds to the population of its *S_A_* but, since the pyrene GSB immediately appears, the excited population is instantly shared with the acceptor. In this sense, the exciplex is formed instantaneously, but the emission stems only from the exciplex state that involves the *S_A_* state of the populated **P**. A pictorial representation of the photocycle of the four systems is reported in Figure 3. This model explains the efficiency of the ET process and the delayed appearance of the exciplex emission.

The investigations of monomers **α** and of the MCS1‐A (Figure S8 and Figure S9, respectively) provide an identical picture compared to the analogous C‐linked systems, namely **P** and MCS1‐C. This proves that the linker substitution, despite the significant changes in the optical spectra, has no effects on the donor photophysics even in the stacked configuration. For the sake of completeness, the relative detailed analysis and discussion is reported in the Supporting Information. More relevant to this article is the study of the system in presence of the acceptor, MCS2‐A. Figure 1h summarizes the TA measurements on MCS2‐A upon excitation of **α** at *λ*_exc _= 327 nm. We observe the instantaneous appearance of pronounced negative signals at *λ* < 350, 370, 390, and 450 nm on top of a broadband positive ESA. The comparison with the steady state spectra (inverted spectra in Figure 1h), confirms that the negative signal at *λ* < 350 nm is mainly due to **α**’s GSB; the two negative peaks at 370 and 390 nm and the band at 450 nm are assigned to GSB and SE, respectively, from excited **Y**. This definitively proves the instantaneous (within tens of fs) transfer of part of the excitation energy to the acceptor **Y**.

Thus, the population of the acceptor is tracked down by measuring the rise of the **Y** GSB signal. Let us assume that the amplitude of the **Y** GSB signal (6 mOD at 390 nm) for a delay of 300 ps corresponds to a complete energy transfer from all **α**s as evidenced by the simultaneous decay of **α**s GSB at *λ* < 350 nm. By comparing this amplitude to that of the GSB of **Y** at 150 fs (4 mOD) in Figure 1h, we find an overall increase of the initial **Y** signal by ca. 50%. This leads to two key results: first, 2/3 of the **Y**s are excited within the pump pulse duration, which is definitively more than the maximum possible ET transfer from the two **α**s closest to **Y**. Second, the remaining **Y** signal increases within the entire measurement time window (0–400 ps) and is accompanied by a recovery of GSB at *λ* < 350 nm. The former confirms that the coherent ET mechanism due to higher delocalized *S_B_* state still occurs, while the latter proves that the reminder of the excited population localizes on one of the **α**s and eventually is transferred to **Y** via a slower ET mechanism spanning ps to hundreds of ps.

The data analysis in Figure 1i gives us more information on the spectral evolution of the excited states. *τ*_1_ is a weak pulse limited signal which resembles the first component in Figure 1f. Accordingly, it describes mainly a spectral relaxation of ESA at *λ* > 470 nm and a fast decay of SE from higher excited states of the photoexcited **α**. *τ*_2_ mainly describes the decay of populated ESA originating from excited state absorption of **α** and the rise of **Y** GSB signal and its SE. In consistence with the study of the **P** monomer and MCS‐C, we assign the former to the departure from the FC region and the latter to IC from delocalized higher *S_B_* to the low‐lying localized *S_A_* state. The next DASs, with time constants *τ*_3 _= 0.75 fs, *τ*_4 _= 3.3 ps, *τ*_5 _= 33 ps and *τ*_6 _= 200 ps, all show the double peak signature of the **Y** absorption at 370 and 390 nm and a negative amplitude at *λ* < 350 nm (inset of Figure 1i), where the **α** absorption is located (black dashed inverted spectrum in Figure 1h). Accordingly, they mainly describe a rise of **Y** signal accompanied by a ground state recovery of the **α** ground state, revealing a multiphasic ET. Following these assignments, we rename τ_1_ and τ_2_ as τ_*FC*_ and τ_*IC*_, whereas τ_3_ to τ_6_ as $\tau_{1}^{ET}$ to $\tau_{4}^{ET}$.

**Figure 4:**
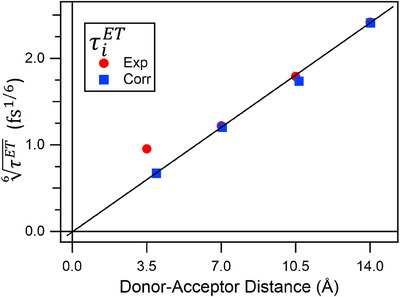
Experimental energy transfer (ET) time constants ($\tau_{i}^{ET}$) as a function of the acceptor–donor distance, calculated as multiple of 3.5 Å. The same experimental data corrected for the mutual geometrical displacement of the chromophores (Table S3 and relative discussion) are shown. Data are plotted in a 6th–root, line scale and the black line represent a pure *R^6^* law with zero intercept.

The $\tau_{i}^{ET}$ show a characteristic *R_i_* ^6^ behavior (Figure 4), where *R_i_* is the distance between the acceptor and the ith donor, approximated by an integer multiple of 3.5 Å, according to X‐ray diffraction studies^[^ ^25^ ^]^ and MD studies^[^ ^26^ ^]^ on similar systems. This dependence is characteristic of Förster Resonance ET (FRET, Equation S3) and is consistent with the observed slow ET channels. Indeed, when corrected by the mutual displacement of the chromophores, the agreement with a *(R/R_0_*)*^6^* law is excellent, where the Förster distance, *R_0_*, is estimated to be (26.9 ± 0.9) Å (see Supporting Information for details on the calculations).

**Figure 5:**
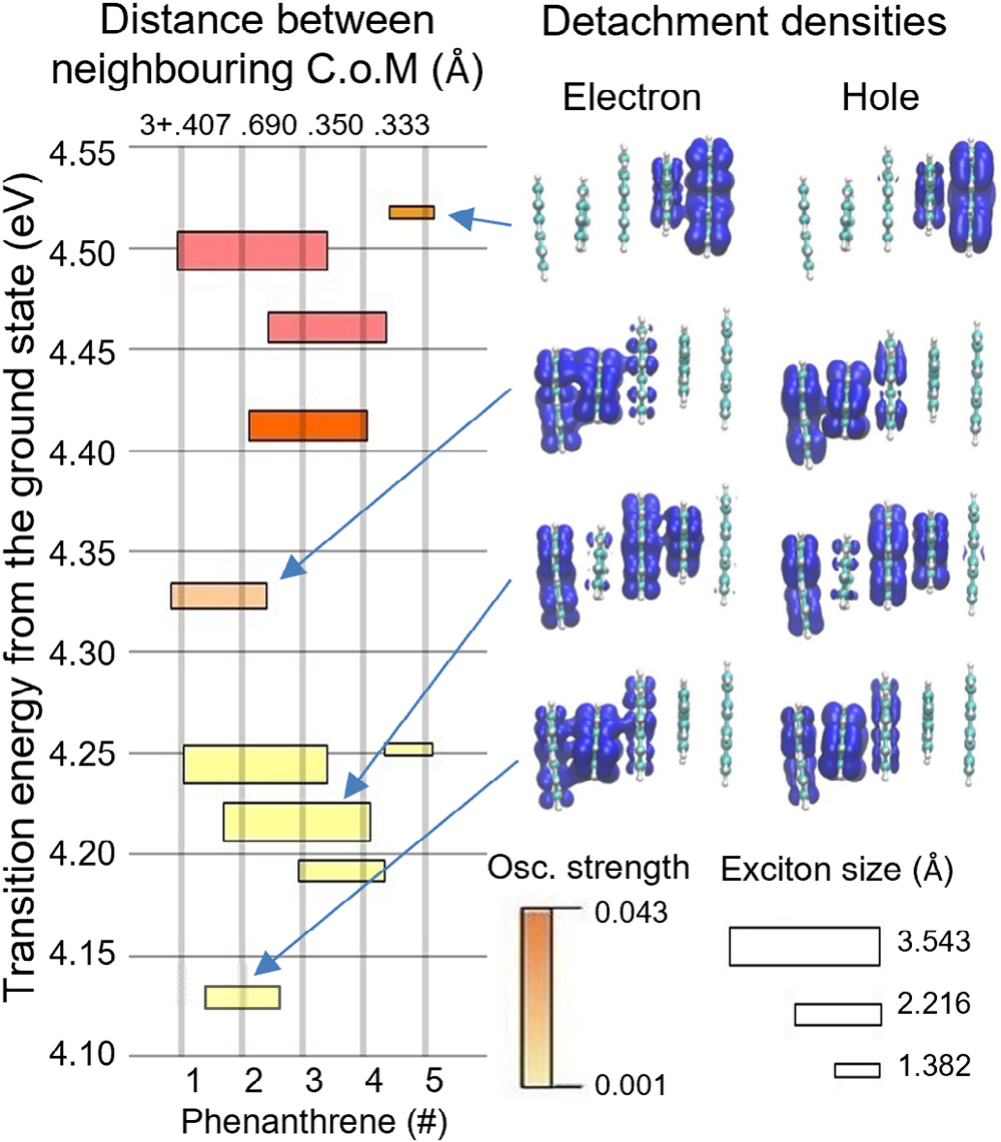
Energy level diagram and visualization of exciton delocalization for the first ten excited states of the stacked phenanthrene pentamer in helicoidal conformation (C.o.M = centre of mass). The positions of the polymer units are marked by grey vertical lines, whereas the exciton positions are given by the box centers. The oscillator strength color code and the size code for the exciton extension are shown in the bottom‐right panel. Representative detachment (electron) and attachment (hole) densities are shown. The results for the parallel and antiparallel configurations are reported in Figure S12.

According to these outcomes, we propose the model in Figure 3 to explain ET in MCS‐A: the presence of delocalized *S_B_* state is unaffected by the linker; the lack of exciplex formation weakens the acceptor–donor coupling and allows for a competitive branching toward the localized state (*S_A_*) of one of the **α**; while 2/3 of the excitation energy is transferred from the delocalized states to the acceptor by coherent ET (τ_*coh*_ ≈  100 fs), the remaining 1/3 is transferred from the localized states *S_A_* by FRET with different rates depending on the **α**‐**Y** distance. The fact that we observe in Figure 1 all the possible FRET time constants with comparable amplitudes (at least the first three) points to a nonselective population of the donors after the branching. This agrees with the linear dependence of the acceptor emission QY on the length of the phenanthrene array.^[^ ^1^ ^]^ This model is very similar to that for MCS‐C. The main difference is that in the latter the AcD coupling is so efficient that the ET can occur only in the delocalized state *S_B_*, making the fraction of donors populating the localized states *S_A_* negligible. In addition, in the MCS‐A the coherent ET should happen only from the delocalized state of equilibrated *S_B_* since the fraction of acceptors populated before the branching should be negligible. This also allows for a complete delocalization of the excitation over the whole stack as confirmed by the nonselective population of the donors after the branching. This is pictorially represented by the thick red arrow denoting energy delocalization which is now two sided in Figure 3. To corroborate the picture inferred from the spectroscopic data and to get a deeper insight into the involved electronic states, and particularly into the presence of delocalized states that could sustain a coherent energy transfer (CET) process, we calculated the electronic properties of a simple model consisting of five π‐stacked phenanthrene units. In the following, consistent with the native quantum chemistry results, we will use an adiabatic notation for the excited states (S_0_, S_1_, and S_2_, …) based on energy order. This can be related to the *S_A_* and *S_B_* diabatic notation used previously, which was based more on the nature of the states and the electronic density reorganization. It is worth noting that the general notation of *S_A_* and *S_B_* inferred from time‐resolved data could also mask a manifold of degenerate or quasi‐degenerate adiabatic states S_n_. To minimize the computational effort and avoid any spurious effects due to the conformational flexibility, we discarded the peripheral substituents keeping only the aromatic cores. This also permits to concentrate on the inherent electronic properties of the MCSs. We manually built three configurations composed of a parallel and antiparallel arrangement, as well as a helical structure that can be seen as intermediate between the formers (Figure S12). The ground state electronic structure has been solved at density functional theory (DFT) level while its time dependent (TD‐DFT) extension is used for the excited states. Because of the conformational constraints exerted by the DNA scaffold, the experimental configuration can only lie between antiparallel and helical arrangements. Since both originate from a similar potential energy surface topology, we will discuss mainly the latter (Figure 5). The exciton delocalization is pictorially shown via the size of the box, and the oscillator strength via the corresponding color code. The parallel and the antiparallel configurations are discussed in detail in the Supporting Information.

We identify a manifold of bright states at 4.33–4.52 eV, consisting of a higher localized state (mainly **P**5) and low‐lying extended ones (over two and three **P** units). A second manifold of delocalized states is found at 4.19–4.25 eV, whereas the lowest excited state S_1_ (4.13 eV) shows a strong localized character basically over one phenanthrene only (**P**2). These observations are also confirmed by the attachment (AD) and detachment (DD) densities, reported in Figure 5 for few representative states. AD and DD represent the electron and hole densities, respectively, and thus identify those regions of the molecule on which the electron density is accumulated (attachment) or retrieved (detachment) upon electronic excitation. It is important to note that we cannot observe a full delocalization due to boundary effects inherent to the finite system and due to the algorithm used for the topological density analysis, which is based on projection of density and hence tends to slightly underestimate the number of delocalized units.

Comparing the parallel and the antiparallel configurations (Figure S12) reveals that the spatial extension of the delocalized states in the bright manifold dramatically depends on the type of assembly, whereas the delocalization character of the low‐lying manifold is robust against changes of the assembly, geometry linking, antiparallel, and helical arrangements.

Noteworthy, the parallel configuration shows a smaller amount of delocalization, indicative of the importance of alternating the phenanthrene arrangements to promote delocalization. Concerning the localized character of S_1_, its existence cannot be seen as a computational artefact because we optimized the MCS from an ideal symmetric situation, i.e., artificially favoring delocalization. This state is mainly localized over one phenanthrene because its excitonic size (2.2 Å) is smaller than the interchromophore distance (3.5 Å). This also agrees with the *R* ^6^ dependence of FRET lifetimes, which speaks against the excitation being delocalized over more donors. Noteworthy, since we observe no change in the emission of MCS1 in Figure 1, we can exclude any **P**‐**P** or **α**‐**α** excimer formation. Considering the natural flexibility of the system and that, very likely, it fluctuates between helical and antiparallel geometries, we argue that the bright states give mainly origin to localized transitions randomly distributed over the whole stack. Also, we expect that such a mixed character facilitates delocalization already during the initial internal conversion.

After photon absorption by one of these local, i.e., monomer‐like, states, the system evolves by internal conversion into the low‐lying manifold of delocalized states that span over almost the entire stack and is instrumental to allow for the long‐range coherent ET. Eventually the system relaxes to the localized and monomer‐like lowest excited state S_1_. This scenario is essentially identical to the one inferred from the experimental results and provides a consistent rationalization of the observed spectral features. We assign the higher bright manifold as the just excited *S_B_* states (the Frank–Condon region), the low‐lying manifold as the relaxed *S_B_* states and S_1_ as *S_A_*. Obviously, the exact unit on which the emissive S_1_/*S_A_* state will localize depends on the differences induced by thermal disorder that could change the exciton localization. More importantly, the photocycle including absorption to a localized state, delocalization, and subsequent relocalization to an emissive state, is found both for antiparallel and helical conformation. This suggests the robustness of these features that should persist even in presence of thermal motion, as actually observed experimentally.

**Figure 6:**
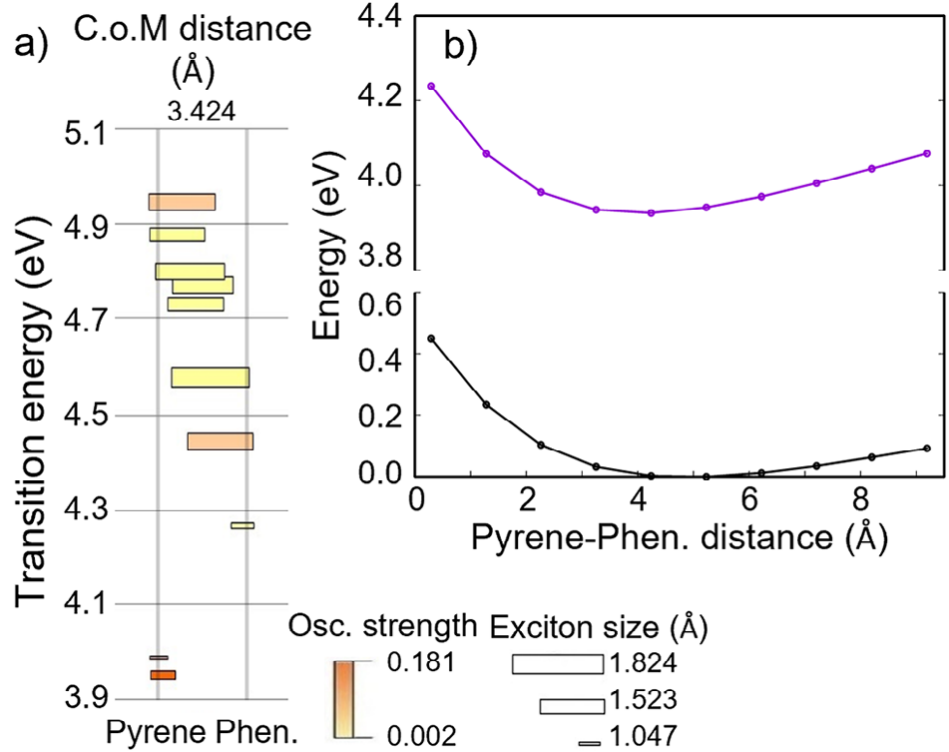
a) Energy diagram and characterization of the excitons for a pyrene/phenanthrene dimer (see caption of Figure 5 for notation). b) Potential energy surface along the interchromophore distance for the ground state and the first excited electronic states.

Finally, to get more insight on the phenanthrene/pyrene ET phenomena we also modelled a stacked dimer in the same three configurations (Figure S13). The most relevant case of the helicoidal arrangement is reported in Figure 6. Differently from the stacked phenanthrenes’ system, this time the overall outcome does not depend on the specific orientation, in agreement with the more symmetric geometry of the pyrene (Scheme S1). Unsurprisingly the lowest excited states lie on the pyrene units (Figure 6a), while a manifold of excited states is present in the 4.2–4.5 eV range, i.e., the region spanned by the delocalized excitonic states of the phenanthrenes stack (Figure 5). This includes a state delocalized over the two units and a second state centered on the phenanthrene. It is evident that the former can act as a funnel, not only driving the energy toward the pyrene moiety but also backward due to a branching of the wavepacket. Furthermore, the equilibrium interchromophore distance is shortened by ∼1 Å in the excited electronic state as compared to the ground state (Figure 6b). This is consistent with the excimer formation and the observed redshift of pyrene emission in the presence of phenanthrenes (see Figure S1B and discussion). This fact is also consistent with the spectroscopic observations indicating that the initial population of the lowest‐lying excited state is followed by excimer formation. Although the internal conversion leading to the population of the excited state through the delocalized manifold happens on ultrafast time scales, the geometrical reorganization, i.e., the reduced spacing between the two conjugated moieties, could last picoseconds, in particular when embedded in MCSs.

## Conclusion

In this contribution we unravel the origin of the reported efficient ET over several nanometres (tens of nm in self‐assembled analogues) with unitary quantum yield^[^ ^1^, ^3^ ^]^ in artificial light‐harvesting phenanthrene antennas embedded in a DNA duplex. By means of fs‐TA spectroscopy and TD‐DFT calculations, we show that the fundamental mechanism is fully quantum coherent. Observing such a process over so long distances is already remarkable, since excited states in large aggregates of closely packed molecules can be easily quenched, thereby suppressing long‐range energy migration.^[^ ^4^, ^5^, ^18^, ^27^ ^]^ What makes the current result exceptional is that the long‐range coherent ET is an anti‐Kasha process.^[^ ^19^ ^]^ Indeed, the electronic states that mediate the ET are not the lowest but higher excited states, which are relatively short‐lived and optically accessible.

Without acceptor, the population in these high‐lying delocalized states persists for almost 1 ps before relaxing toward low‐lying excited states. These states cannot sustain any coherent ET since they are localized around individual phenanthrenes.

The insertion of an acceptor makes the ET site specific, and as a result, the energy is coherently localized on the acceptor in a few tens of fs. Also, we observed that ET from the antenna to the acceptor can be enhanced by at least one order of magnitude by choosing an acceptor capable of forming an exciplex with the closest donors.

We investigated two variants of MCSs, in which the acceptor can either form or not form an exciplex with the excited closest donors. In the former case we observed a pulse‐limited (< 40 fs) fully coherent ET only from the light‐harvesting stack to the acceptor. This ET is so efficient that it overcomes any internal conversion toward the lowest singlet excited state, making it a fully anti‐Kasha coherent energy transfer process. Interestingly, even if the energy localization is instantaneous, within the experimental time resolution, the formation of the emissive state of the exciplex takes ∼ 0.5 ps. In the latter case, only a fraction of the excitation energy is transferred coherently, while the remaining part relaxes into the lowest singlet excited state, which has a strong localized character around one of the chromophores. Ultimately, the energy is still transferred to the acceptor but incoherently via a Förster resonance energy transfer.

We conclude that the exceptionally high ET QY relies on the interplay between bright, short‐lived, high‐lying delocalized states and dark, long‐lived, low‐lying localized states connected via conical intersections. The effect of the linkers on the donor–donor coupling is negligible and the main mechanism of energy redistribution among the phenanthrenes is always coherent ET. However, to fully exploit such systems in terms of light harvesting the coupling between the donors’ stack and the acceptor is crucial, since delocalization occurs on higher‐lying states and therefore competes with IC processes. In this regard, the formation of an exciplex between the closest donor and the acceptor plays a key role to achieve efficient light harvesting, since it can enhance the donor to acceptor ET up to the point to be competitive with IC processes.

## Conflict of Interests

The authors declare no conflict of interest.

## Supporting information



Supporting Information

## Data Availability

The data that support the findings of this study are openly available in [https://boris.unibe.ch/] at [10.48350/171106], reference number [171106].
