# Supplementary material for: Long Range Coherent Energy Transfer in Artificial Multichromophoric Antenna Systems—A Case of Breaking Kasha's Rule
Source: Angew Chem Int Ed Engl. 2025 Aug 1;64(38):e202513001. doi: 10.1002/anie.202513001 (PMC12435419; doi:10.1002/anie.202513001)
Supplement: Supplementary file 1 — Supporting Information [file ANIE-64-e202513001-s001.docx]

**Long Range Coherent Energy Transfer in Artificial Multichromophoric Antenna Systems – a Case of Breaking Kasha's Rule**

M. Nazari Haghighi Pashaki^1^, C. D. Bösch^2^, F. Garo^2^, A. Blanc^3,^ M. Marazzi^4,5^, A. Rondi^1^, M. Gazzetto^1^, M. Akbarimoosavi^1^, J.-C. Tremblay,^3^ S. M. Langenegger^2^, A. Monari^3,6^, R. Häner^2^, T. Feurer^1^, A. Cannizzo^1^

^1^ Institute of Applied Physics, University of Bern, Switzerland

^2^ Department of Chemistry, Biochemistry and Pharmaceutical Sciences, University of Bern, Switzerland

^3^ Université de Lorraine & CNRS, LPCT UMR 7019 F-5400 Nancy - Metz, France

^4^ Universidad de Alcalá, Departamento de Química Analítica, Química Física e Ingeniería Química, Functional Molecular Systems (FuMSys) group, Ctra. Madrid-Barcelona Km. 33,600, E-28805, Alcalá de Henares (Madrid), Spain

^5^ Universidad de Alcalá, Instituto de Investigación Química "Andrés M. del Río" (IQAR), Ctra. Madrid-Barcelona Km. 33,600, E-28805, Alcalá de Henares (Madrid), Spain

^6^ Université Paris Cité and CNRS, ITODYS, F-75006, Paris, France

**SUPPLEMENTARY INFORMATION**

Contents

SI.1 Materials and Methods 12

SI.2 Further steady state absorption and emission spectra 15

SI.3 Evidence of no α‑Y excimer formation in MCS2-A: steady state emission of alkynyl-pyrene in different assemblies 17

SI.4 Transient absorption spectroscopy on carboxamide phenanthrene monomer (P) 19

SI.5 Comparison between carboxamide phenanthrene monomer (P) and MCS1-C 20

SI.6 Differential TA spectra of carboxamide MCSs (MCS1-C and MCS2-C) 22

SI.7 Ultrafast transient absorption spectroscopy on alkynyl-phenanthrene monomer (α) and the MCS1-A 23

Alkynyl-phenanthrene monomer 23

Multichromophoric systems with alkynyl phenanthrenes without acceptor (MCS1-A) 23

SI.8 FRET calculations 25

SI.9 Energetic levels and exciton delocalization for phenanthrene pentamer and the pyrene‑phenanthrene dimer in parallel, helix and antiparallel conformations. 28

SI.10 Comment on the coherent nature of the energy transfer mechanism 31

References: 31

# Materials and Methods

**Excitation rate in MCS.** To fully understand the relevance of $\varepsilon>{10}^{6}$ M^‑1^cm^‑1^ and an ET range of 10s of nm we should consider the typical sun light intensity as a reference. Sunlight intensity at zenith at 42° latitude corresponds to approximately 1 visible photon/nm^2^ each 200μs and, with some collecting optics (focusing 1 cm^2^ into 100 μm^2^), values of 1 photon/nm^2^ each 10 ns or less can be achieved. Under such fluence conditions, a dye with a typical extinction coefficient of 10^4^‑10^5^ M^‑1^cm^‑1^ would have a probability to be excited once each 2-0.2 μs while a molecular system with $\varepsilon\sim{10}^{6}$ M^‑1^cm^‑1^ would be excited each 20 ns (or the probability that a photon is absorbed by one molecule is 5% and 50%, respectively).

**Sample.** The investigated systems are DNA-guided MCSs with phenanthrene building blocks as donors and a pyrene moiety as an acceptor placed in a DNA duplex.^[1]^ Chemical structures are shown in Figure 1 of the main article, and in Table S1 and Scheme S1. The incorporation of non-nucleotide moieties in the DNA backbone requires the functionalization with suitable linkers. In this study, we investigated systems with two linkers, a flexible carboxamide ^[1,2]^ and a more rigid alkynyl linker, ^[3,4]^ since they can affect the excited state dynamics and ET processes of the MCSs likely altering the π-π interaction between stacked chromophores. Indeed, steady state fluorescence shows that the excitation of phenanthrenes in MCS with carboxamide linkers leads to the formation of an emissive pyrene-closest-phenanthrene exciplex, which is missing in systems with alkynyl linkers. The investigated DNA-guided MCSs are listed in Table S1, where it is also explained the sample notation.

The MCSs were prepared in an aqueous solution of 10 mM sodium phosphate buffer (pH 7.0, 100 mM NaCl). The concentration was ~50 μM, which is enough to have an OD at λ_exc_ between 0.1 and 0.3 in 200 μm light path.

Samples were characterized by mean of steady state absorption (Lambda 750 by PerkinElmer) and emission (Cary Eclipse spectrofluorimeter from Agilent) spectroscopies (see Figure S1). Steady state absorption (OA) spectra before and after transient absorption measurements were measured and no sign of photodamages was observed.

Table S1. Investigated multichromophoric systems (MCSs). Each MCS is a duplex of two single strands containing adenine (A), thymine (T), carboxamide-linked phenanthrene and pyrene (**P** and **S**, respectively) and alkynyl-linked analogues (**α** and **Y**, respectively). See Scheme S1 for the molecular structures. For sake of readability, MCS with and without the acceptor are labelled “1” and “2”, while the suffixes –C and –A refer to MCSs with carboxamide and alkynyl likers, respectively. When the labels or the suffixes are not specified (*e.g.* MSC‑C or MSC2) we refer to both the respective MCSs (*e.g.* MSC‑C stands for MSC1‑C and MSC2‑C, whereas MSC2 for MSC2‑C and MSC2‑A).

| Systems | Formula |
| --- | --- |
| MCS1-C | 5’ TAA TAA ATT **PPPPP** TTA AAT AAT  3’ ATT ATT TAA **PPPPP** AAT TTA TTA |
| MCS2-C | 5’ TAA TAA ATT **PPPPP** TTA AAT AAT  3’ ATT ATT TAA **PPSPP** AAT TTA TTA |
| MCS1-A | 5’ TAA TAA ATT **ααααα** TTA AAT AAT  3’ ATT ATT TAA **ααααα** AAT TTA TTA |
| MCS2-A | 5’ TAA TAA ATT **ααααα** TTA AAT AAT  3’ ATT ATT TAA **ααYαα** AAT TTA TTA |


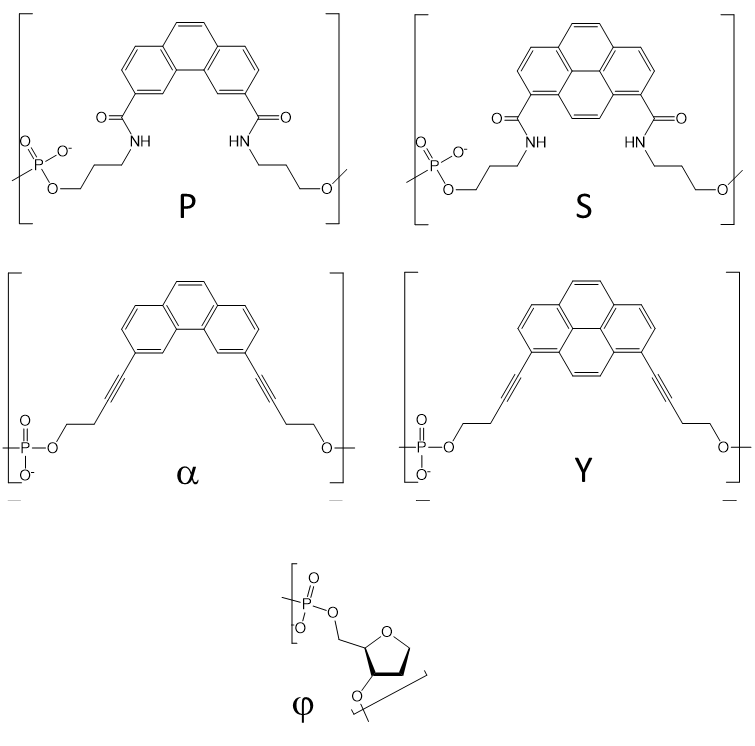


Scheme S1. (Top) molecular structures of the carboxamide-linked phenanthrene (**P**) and pyrene (**S**); (Bottom) alkynyl-linked analogues (**α** and **Y**, respectively).

**Transient absorption spectroscopy.** For detailed information on the setup we refer to the literature^[5,6]^. About experimental conditions, samples were prepared in an aqueous solution with 10 mM sodium phosphate buffer (pH 7.0, 100 mM NaCl). The concentration was chosen about 1~μM to have an OD of 0.1 ‑ 0.3 in 200 μm light path at the maximum of phenanthrene absorption (320 nm and 327 nm for **P** and **α**, respectively). The sample was flowed using a flow‑cell with 200 μm optical path and pumped with a microgear pump (mzr-2505) to avoid sample accumulation and multiple excitation during TA measurements ^[5]^.

To meaningfully compare the amplitudes of the spectral signals in Figures 1 and 2 of the main article, monomer and MCS2 spectra were normalized to the ones of MCS1 with respect to the number of excited molecules, and excited with identical conditions: excitation at the maximum of **P** or **α** absorption (320 nm or 327 nm, respectively), with 100 nJ/pulse at 2.5KHz. The time resolution was 40 fs standard deviation. Pump and probe polarizations were set parallel.

**Time-Spectrum decomposition analysis.** To extract kinetic and spectral information from the experimental data we carried out a Single Value Decomposition and Global Fit (SVD-GF) analysis ^[7]^ on the transient absorption data $\mathrm{TA}\left( \lambda,t \right)$. In this way we can first separate the noise, $\Xi\left( \lambda,t \right)$, from the spectral evolution of the system, $\hat{TA}\left( \lambda,t \right)$,

| $\mathrm{TA}\left( \lambda,t \right)=\hat{TA}\left( \lambda,t \right)+\Xi\left( \lambda,t \right)$ | eq. S1 |
| --- | --- |

second, we can decompose the latter as an expansion of exponential decays with characteristic lifetimes ($\tau_{k}$) and decay associates spectra (DASs)

| $\hat{TA}\left( \lambda,t \right)=\sum_{k=1}^{k=p} {DAS}_{k}\left( \lambda\right) e_{t>0}^{-\frac{t}{t_{k}}} \vert_{IRF(K_{B},t_{0},t)}$ | eq. S2 |
| --- | --- |

where $e_{t>0}^{-\frac{t}{t_{k}}} |_{IRF(K_{B},t_{0},t)}$ represents an exponential decay with decay constant $\tau_{k}$ multiplied by a Heaviside unit step function $u_{0}(t)$ and then convoluted with an instrumental response function (IRF), supposed Gaussian (in time zero, $t_{0}$, and with a width of $K_{B}$).

**Molecular modelling and simulations.** To simplify the problem and disentangle the fundamental electronic effects we considered only a model system composed of stacked aggregates of the main chromophores, i.e. phenanthrenes and pyrenes. To minimize the computational effort and avoid any spurious effects due to the conformational flexibility, the peripheral substituents were discarded, keeping only the aromatic cores. Three conformations have been considered for a five-membered aggregate of phenanthrenes (pentamer) namely a parallel, antiparallel, and helicoidal arrangement. In addition, a phenanthrene/pyrene dimer has also been considered to get more insight in the acceptor-donor coupling and to model the energy transfer phenomena. In the latter case parallel, antiparallel, and perpendicular conformations have been considered. The geometry of the ground state of each system has been optimized with Density Functional Theory (DFT) calculation, performed using Gaussian 09.^[8]^ The wB97XD^[9]^ range-separated hybrid functional is employed to also take into account empirical dispersion correction, which are necessary to properly model the α-stacking observed in the systems under consideration here. The 6-31G(d) basis set is used consistently for all the calculations. The excited states characterization is performed via Time-Dependant Density Functional Theory (TD-DFT) using Gaussian 16. For this purpose, the hybrid exchange-correlation functional CAM-B3LYP^[10]^ is used to provide a good description of possible charge-transfer states, while the 6-311+G(d) basis set is used to assure a sufficient flexibility for the electronic density reorganization. The excited state densities, and in particular the exciton features, such as its size and localization, are analysed for each excited state via the Theoretical Density, Orbital Relaxation and Exciton analysis (TheoDORE) package.^[11]^ TheoDORE uses the cclib Python library^[12]^ to perform a Löwdin population analysis of the transition density matrices yielding the topological descriptors reported in the present work. Detachment and attachment densities for the principal excited states of the helical conformation are reported in Figure 5. They are obtained by a diagonalization and subsequent partition of the difference density matrix between one specific excited and the ground state and represent the electron (attachment) and hole (detachment) densities, respectively. In other terms they identify the region of the molecule on which the electron density is accumulated (attachment) or retrieved (detachment) as a consequence of the electronic transition.

# Further steady state absorption and emission spectra

The steady state absorption and emission studies (Figure S1) show that the optical absorption of **P** stacked in MCS‑Cs is very similar to the **P** monomer, with the absorption maximum centred at 320 nm. As reported in previous studies,^[1,2]^ it scales linearly with the number of incorporated **P**. The presence of the **S** moiety makes an additional absorption band appear at 360nm, where the expected fingerprint of the acceptor is located. **P** in DNA-guided stacks show a characteristic fluorescence at ca. 415 nm, respectively (red dashed lines in Figure S1A).^[13,14]^ Remarkably, insertion of one **S** in a MCS-C of **P**s suppresses the **P** emission and give origin to an emission band at 450nm, which is neither the **P** nor the **S** (green, blue and yellow dashed lines in Figure S1A, respectively). This band was assigned to the formation of an exciplex between **S** and the closest **P**. Previous studies point to a very efficient ET in MCSs from the π-stacked **P**s to the pyrene,^[13,16, 22]^ which is essentially independent on the distance of initially photoexcited **P** and **S** at least over several tens of chromophores.

| 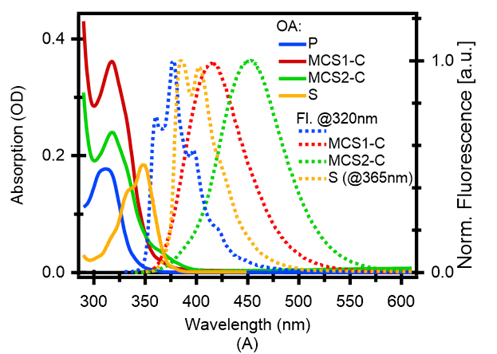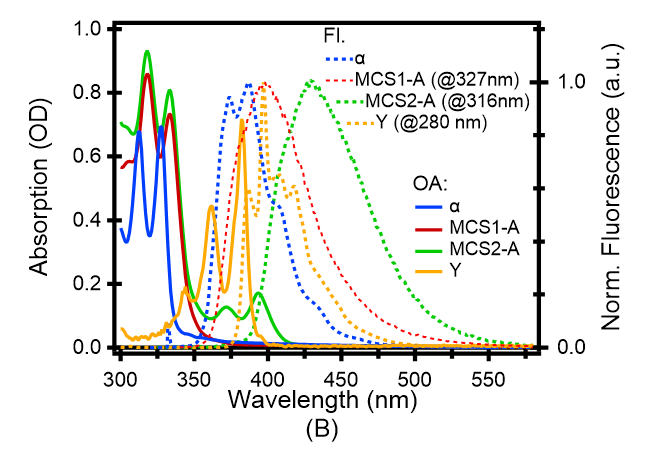  Figure S1. (A) steady state absorption (OA) and normalized emission (Fl.) spectra (solid and dashed lines, respectively) of MCS1-C and MCS2-C. For sake of completeness the absorption and emission of aqueous **P** and **S** monomers (blue and yellow lines, respectively) are shown. (B) Analogous steady state measurements of aqueous **α**, **Y**, MCS1-A and MCS2-A. Emission were excited at 320 nm, if not differently specified. The reported absorption spectra are the ones of the solutions used for the transient absorption experiments (optical path 200 μm). The absorption spectrum of the S monomer was collected in a 1 mm optical path cuvette, because it is much less soluble than the other systems. |
| --- |

Moving to alkynyl linked chromophores, both **α** and **Y** show a more structured absorption with respect to their carboxamide counterparts, with two clear vibronic replica (313/327 nm and 372/394 nm for **α** and **Y**, respectively (Figure S1B).^[15]^ Stacked systems (MCS1-A and MCS2-A) show the same OA spectrum and very similar (slightly red shifted) to the one of the monomer **α**, except for the presence of two peaks at 372 nm and 394 nm in the MCS2-A due to **Y** moiety. Concerning emission, upon excitation at 327 nm MCS1-A shows a fluorescence band (dashed red line in Figure S1B) assigned to staked **α**, which is completely quenched by the insertion of **Y** in the MCS2-A. The presence of **Y** emission at 430 nm^[4]^ upon excitation of **α** (dashed green line in Figure S1B) confirms that the quenching mechanism is still energy transfer but, unlike MCS2-C, this emission stems from **Y** only because **Y** in MCS2-A does not form any exciplex. Indeed, the emission of **Y** is substantially the same both as monomer or embedded in single strands^[16]^ and duplexes,^[17]^ with and without **α**s (see for instance the comparison in Figure S3). In particular, the fact that the vibrionic progression is the same and the zero-phonon line undergoes a limited shift definitively reveal that the potential energy surface is the same. This proves that the emitter is structurally the same and hence no exciplex is formed. The fact that in the presence of **α**s we observe a more symmetric Franck‑Condon progression speaks for an increase of the relative position of the ground and excited energy surfaces of the **Y**, pointing to an attractive interaction between the excited **Y** and the closet **α**. Since **Y** absorption overlaps with the **α** emission, the ET mechanism is rationalized as Förster Resonance Energy Transfer.^[18]^

To rule out possible artifacts due to potentially directly excited acceptors upon excitation at 320/327 nm, we show in the following that this fraction is negligible and that observed acceptor GSB is only induced by the donor photoexcitation. In case of Y, we can immediately exclude it because the absorption at 327 nm is not detectable (Figure S1B), therefore in the following we will discuss only the carboxylic systems.

Also in the case of **S**, we can already qualitatively see that the possibility to have a direct excitation is negligible, because the ratio of acceptors vs donor is 9 to 1 and the S absorption at 320 nm is already rather small (*ca.* ¼ with respect to its maximum). This is shown in Figure S2, where the absorption of the acceptor (yellow lines) is compared with the absorption of the MCS2‑C (green line).

| 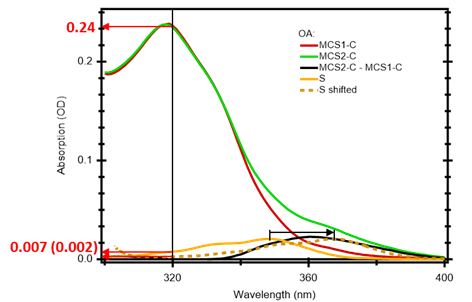  Figure S2. Comparison of the steady state absorption (OA) of MCSC and the acceptor S (see text for more details). |
| --- |

To perform a quantitative comparison, we calculated the contribution of the S absorption to the MCS2‑C one by subtracting from the latter the absorption of the MCS1‑C (red line) scaled to match the maximum of the MCS2‑C absorption at 320 nm. This curve (black line) was used to scale the amplitude of the S band, as reported in the figure (solid yellow line). The amplitudes at 320 nm of the MCS2‑C and S band are 0.24 OD and 0.007 OD, respectively, give an overall fraction of excited acceptors of 3%. This is actually a conservative estimation because the comparison of yellow and black lines reveals the occurrence of a red shift of 18 nm when the acceptor is inserted in the MCS. This is confirmed by the comparison of the absorption and emission spectra of the acceptor Y as monomer or embedded in the MCS2‑A (Figure S1A and Figure S3), where the clear vibronic progression allows an immediate identification of the shift. This suggests that a more realistic comparison needs to apply a red‑shift of 18 nm to the S band (dashed yellow line). After this correction the amplitude of the acceptor absorption at 320 nm is 0.002 OD, which gives a fraction of excited acceptors of 0.8%. The observed GSB amplitude is not compatible with such a low direct excitation yield of the acceptor.

Another point that should not be underestimated is the consistency with the observation on the alkenyl counterparts. Indeed, this assignment is also confirmed by the comparison with the analogous alkenyl MCS2‑A, which show comparable amplitude of GSB absorption (early spectra in the Figure 1i of the main article), without any doubt that the acceptor was not excited (figure S1B). If the spectral features of the acceptor observed on the carboxylic systems would be mainly due to a direct excitation, we should not see this signature of the Y acceptor in the MCS2‑A systems.

# Evidence of no α‑Y excimer formation in MCS2-A: steady state emission of alkynyl-pyrene in different assemblies


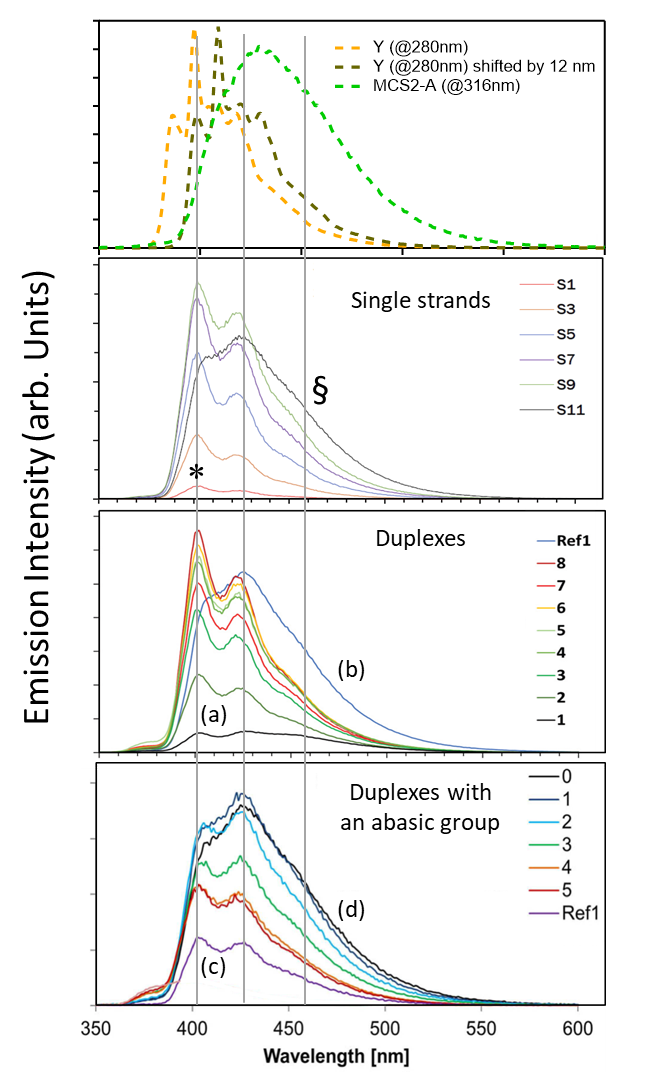


Figure S3. Comparison of emission from alkynyl-pyrene (**Y**) in different assemblies. Top panel shows the emission from monomer and the MCS2-A upon 280 nm and 316 nm excitation (from Figure S1), respectively. A monomer emission red-shifted by 12 nm is also plotted. Emissions at the same excitation wavelengths from single strands and from duplexes assemblies containing one **Y** and a variable number of **α**s are shown (Reproduced from ^[16]^ with permission from the Royal Society of Chemistry, copyright [2020].). The signal marked with * originates from a **Y** hosted in a single stand without alkynyl-phenanthrenes (**α**s). For the sake of completeness, the bottom panel shows emission upon 316 nm excitation in different duplexes where an abasic group is inserted in front of the **Y** to increase the duplex thermal stability (reproduced from ^[17]^ with permission from the Wiley, copyright [2019]).

Figure S3 compares the emission stemming from alkynyl-pyrene (**Y**) in different assemblies. The comparison with the monomer emission (top panel) confirms that the main effect of the DNA-environment is to induce a redshift, whereas the vibrionic progression is the same. Considering the assembly with the highest number of **α**s (§ in second panel, four **α**s) we can still recognize the main features of the **Y** emission. The comparison with signals from duplexes leads to the identical conclusions: regarding the number of **α**s (1 in (a) and 9 in (b), third panel) the **Y** emission is clearly identifiable. For the sake of completeness, the bottom panel shows emission upon 316 nm excitation in different duplexes where an abasic group is inserted in front of the **Y** to increase the duplex thermal stability (reproduced from ^[17]^ with permission from the Wiley, copyright [2019]). Again, the characteristics emission from **Y** is clearly observed even in the presence of **α**s (2 in (c), 8 in (d), bottom panel). Concerning the other signals, they originate from different arrangements of the **α**s and DNA bases, which are described in detail in the respective references. The persistence of the **Y** emission features against so many arrangements definitively speaks for the lack of any **α**‑**Y** exciplex formation.

# Transient absorption spectroscopy on carboxamide phenanthrene monomer (P)

| 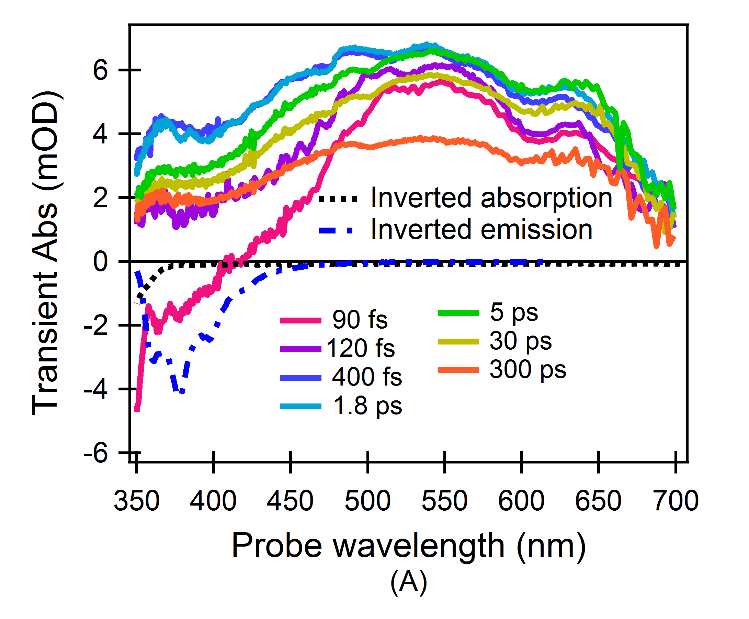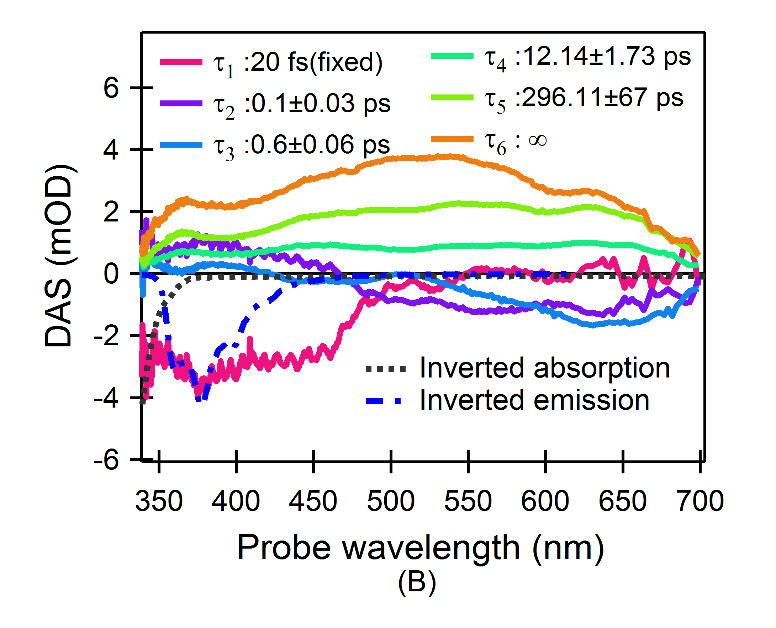 Figure S4. Ultrafast TA spectroscopy on carboxamide phenanthrene monomer (**P**). (A) A representative selection of TA spectra upon excitation at 320 nm. (B) Decay associated spectra and respective decay time constants from the SVD-GF analysis. Reproduced from ^[5]^ with permission from the PCCP Owner Societies, copyright [2019] (Reproduction of material from Physical Chemistry Chemical Physics). |
| --- |

# Comparison between carboxamide phenanthrene monomer (P) and MCS1-C

In this section we compare the Decay Associated Spectra (DASs) reported in Figure 1c and Figure S4B, namely the outcome of the SVD‑GF analysis of the MCS1‑C and the P monomer, respectively.

| **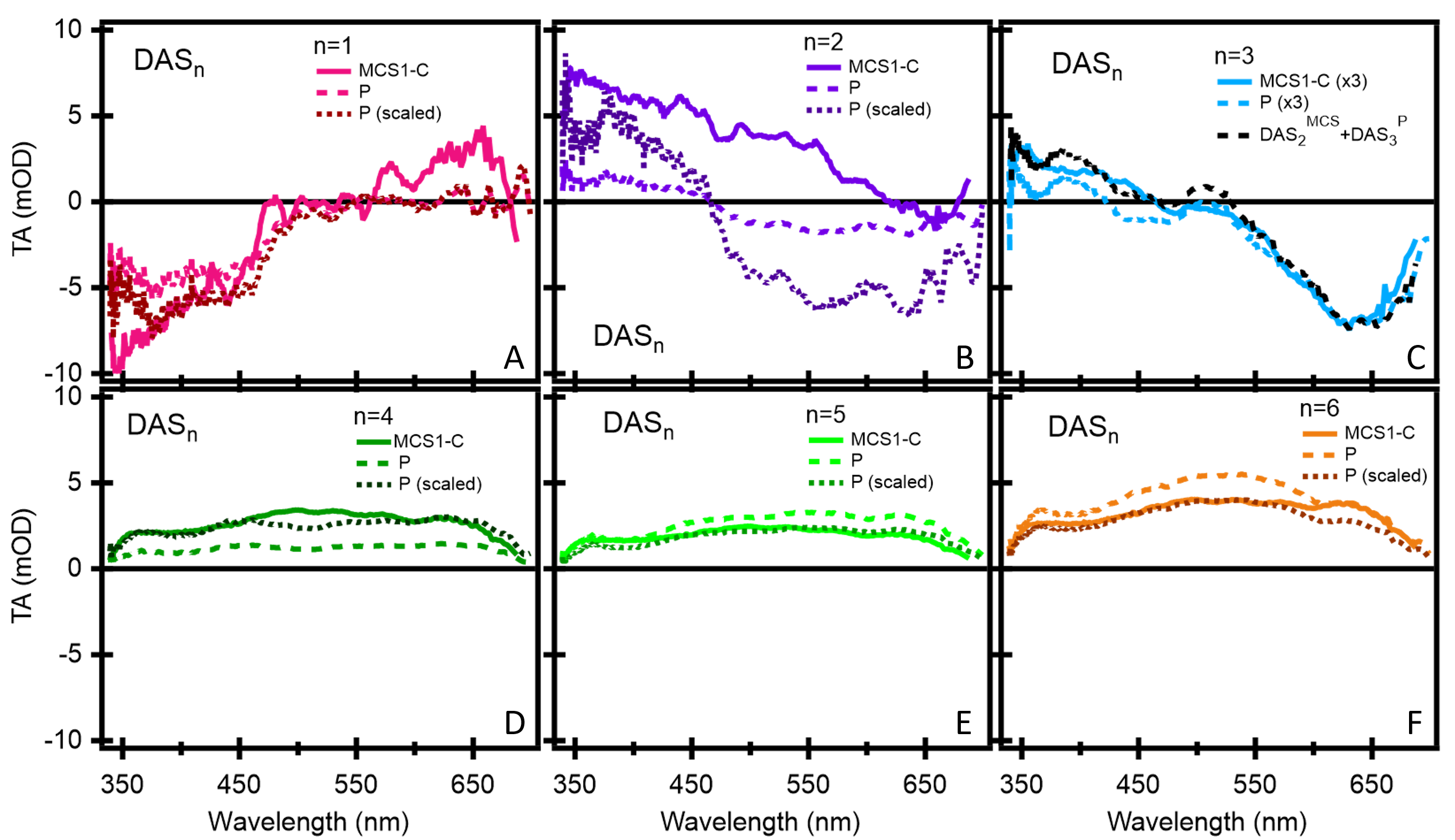**  Figure S5. Comparison of the Decay Associated Spectra (DASs) of the **P** monomer and the MCS1-C from Figure S4 and Figure 1c, respectively (dashed and solid lines, respectively). The amplitudes of the formers are scaled by the same factor to have a comparison consistent with data in Figure 2a-d. A scaled spectrum of the monomer is also plotted (dark, dotted lines) to facilitate the comparison (except for the 3^rd^ component, being unnecessary). The black dashed line in panel C is a linear combination of DAS_2_ of the MCS1‑C (purple solid line in panel B) and the DAS_3_ of the **P** monomer (light blue dashed line in panel C). |
| --- |

The comparison is reported in Figure S5, where the DASs of MCS1‑C (solid lines) are the same in Figure 1c, whereas the ones of the monomer P from Figure S4 (light dashed lines) are scaled by the same factor to match the amplitude of data in Figure 2a-d. To facilitate the intercomparison, we also report monomer DASs scaled to match the amplitude of the respective MCS component DAS (dotted dark lines), except for the 3^rd^ component being unnecessary. All the DASs are very similar except the 2^nd^ and the 3^rd^ ones, which mainly account for the dynamics of the *S_B_* state and the population of the *S_A_*. Considering as a reference the monomer components, both in DAS_2_ and DAS_3_ the effect of the staking can be described as the appearing of a very broad positive signal, rather constant over all the range, superimposed to a monomer-like behaviour. The main feature of the latter is a negative signal at λ > ~500 nm, which describes a red‑shift of the TA signal when the excitation localizes itself on one of the *S_A_* states. In agreement with the study of the **P** monomer, this feature is present in both the 2^nd^ and 3^rd^ DASs pointing to a biphasic IC toward one of the low‑lying states from the unrelaxed and the relaxed *S_B_* state (see ref. [5]). The unstructured and broad signal is dominant in the 2^nd^ DAS of the MCS1‑C ($\mathrm{DA}S_{2}^{\mathrm{MCS}}$) but, at a first glance, it is less evident in the third component ($\mathrm{DA}S_{3}^{\mathrm{MCS}}$), because dominated by the negative feature due to the *S_A_* population. However, the decomposition of the $\mathrm{DA}S_{3}^{\mathrm{MCS}}$ (see black dashed line in Figure S5C) as a linear combination of the $\mathrm{DA}S_{2}^{\mathrm{MCS}}$ and the 3^rd^ DAS of the **P** monomer ($\mathrm{DA}S_{3}^{P}$) confirms that also the signals originating from relaxed *S_B_* are characterized by a unstructured and broad spectrum.

A convenient way to visualize these spectral changes is also to compare the sum of the DASs, $\sum_{n_{0}}^{6} DAS_{n}\left( \lambda\right)$, varying *n_0_* from 1 to 6 (Figure S6) [Frei, F. *et al.* *Dalton Trans.* **2014,** *43* (47), 17666-76.]. To explain the information provided by the comparison of the sums, we should consider the eq. S2. To look at the sums is equivalent to artificially stretch the time constants to have well separated dynamics. Accordingly the sum of all the DAS (red lines in Figure S6A) would be the time-zero spectrum upon a delta-pulse excitation; the sum with n=2 to 6 (purple lines in Figure S6B) would be the spectrum after the departure from the Franck-Condon region and before any relaxation of the *S_B_* state, the third one (light blue lines, panel C) would be the spectrum after the relaxation of S_B_ and the IC from unrelaxed *S_B_*, the forth component (dark green lines, panel D) represent the spectrum after localization on one of the phenanthrenes but before cooling and conformational changes, etc. This is an expedient to clearly visualize the spectral features of each intermediate state (or combination of intermediate states with same time constants) and which kind of dynamics the removed DAS takes into account. However, it should be kept in mind that the observed change is only qualitative since the weight due to the temporal decay is neglected.

Moving to the comparison in Figure S6, we observe that the time-zero spectra (red lines, Figure S6A) are practically identical. This fully corroborates the localized, monomer-like, nature of the initially photoexcited state, as discussed in the main text. The comparison of the second sums (purple lines in the panel B) clearly shows that the spectral features of the *S_B_* state in MCS1‑C, with respect to the monomer, are broader and more unstructured, in agreement with an enhanced delocalized character of *S_B_* and the discussion related to Figure S5. The same analysis holds for the comparison of the third sums (light-blue lines in panel C), despite it is less evident because the amplitude of the third components is much smaller than the others (DAS_3_ in Figure S5 are multiplied by a factor 3). The last three plots show that the long‑lived components from the monomer and the MCS are very similar, corroborating the discussion of Figure 2 in the main text, in particular that the long‑lived emission of the MCS1‑C stems from an electronic density localized on one **P**.

| **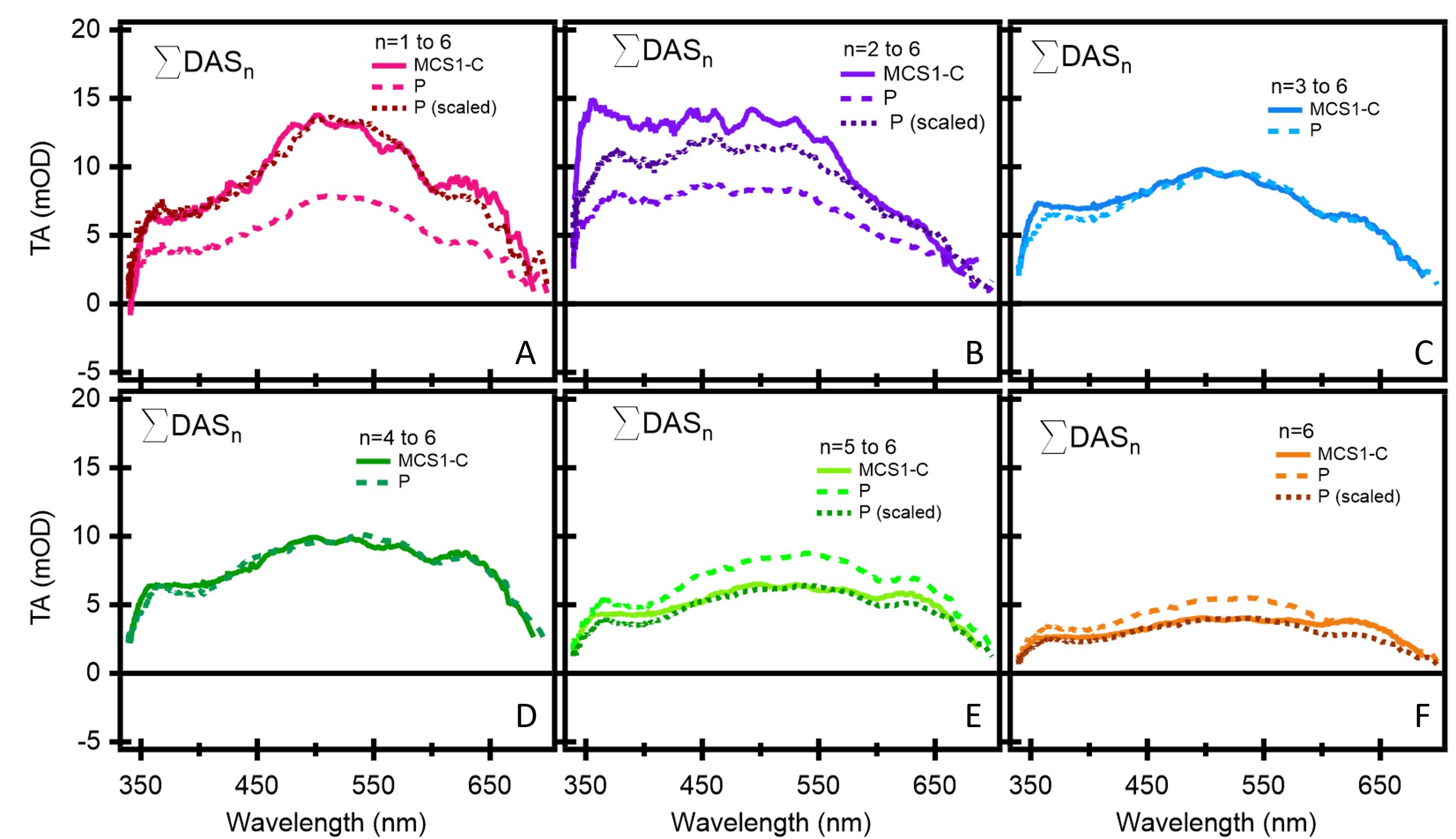**  Figure S6. Comparison of the sums of DAS*_n_*, $\sum_{n_{0}}^{6} DAS_{n}\left( \lambda\right)$, with *n* running from *n_0_* to 6 (*n_0_* = 1 to 6) of the **P** monomer and the MCS1-C (DASs from Figure S5). From left to right and from top to bottom, sums with *n_0_* from 1 to 6. For the sake of completeness, we show the last plot despite is only the DAS_6_ and is the same plot of Figure S5F. The physical meaning of the first sum (panel A) is the time‑zero spectrum deconvolved from the instrumental response function (or, equivalently, excited by a delta pulse). A discussion on the meaning of this sum and the other sums is reported in the text. |
| --- |

# Differential TA spectra of carboxamide MCSs (MCS1-C and MCS2-C)

| 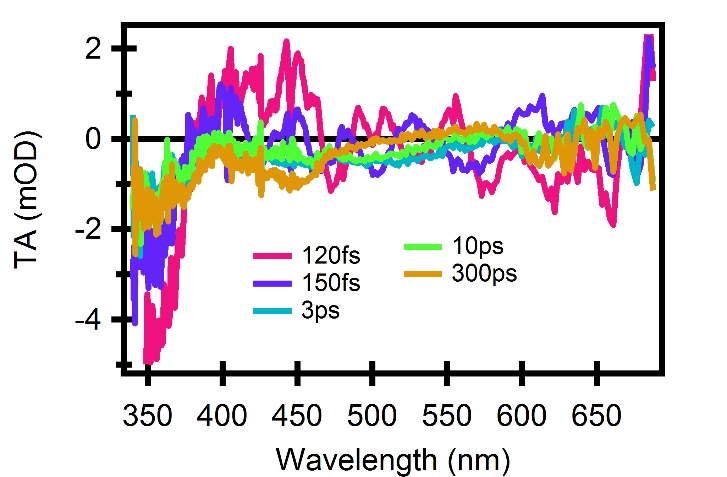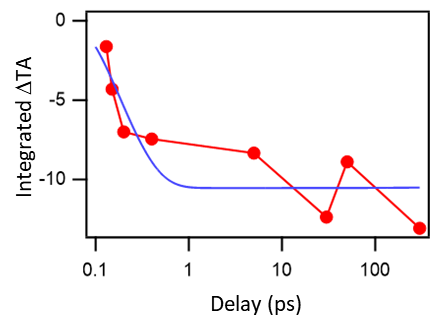  (B)  (A)  Figure S7. (A) Differential spectra of MCS2-C minus MCS1-C extracted from the experimental data in Figure 1. Before subtraction MCS1-C was normalized to maximize the overlap with the MCS2-C signal at λ > 550 nm. (B) Integrated area (red dots) of the spectra from Figure 2e around the maximum of the emission signal (435‑465 nm). The blue solid line is the best fitting of the data with an exponential rise of a negative signal (time constant 0.30 ± 0.15 ps). |
| --- |

As can be seen from the figure. the signal-to-noise ratio of data in Figure 1 is not sufficient to resolve well enough the spectral difference. Therefore, we performed measurements at selected time delays with longer integration time and more averaging to acquire differential spectra with an acceptable quality, which are shown in Figure 2e. These data are consistent with Figure S7 but allow us to identify unambiguously the two negative bands at 360 nm and 450 nm (Figure 2e and relative discussion). To estimate the characteristic risetime the emission at 450 nm (see Figure 2e in the main text and the relative discussion) we plot (red dots) in the right panel the integrated signal around the emission maximum at 450 nm (integration interval 435‑455 nm). The data are fitted with an exponential rise of a negative signal to estimate the emission rise time. The best fitting is obtained with a time constant of 0.30 ± 0.15 ps. It worth noting that, because of uncertainties due to the data quality, the procedure to extract the differential signal, as well the sparseness of data points (see comment to panel (A)) and the choice of the integration range, this value should be considered more as a characteristic risetime of the emission rather than a quantitative estimation.

# Ultrafast transient absorption spectroscopy on alkynyl-phenanthrene monomer (α) and the MCS1-A

## Alkynyl-phenanthrene monomer

| 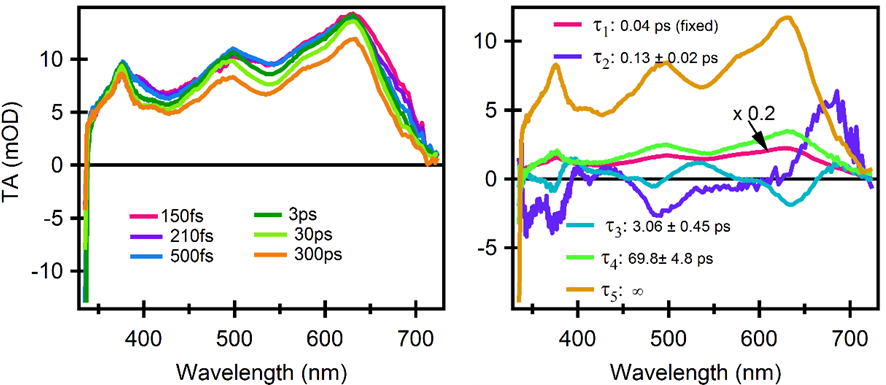  (B)  (A)  Figure S8. Ultrafast TA spectroscopy on alkynyl-phenanthrene monomer (**α**). (A) A representative selection of TA spectra. (B) Decay associated spectra and respective decay time constants from the SVD-GF analysis. |
| --- |

Figure S8A shows the TA measurements on aqueous solution of **α** monomers. Upon excitation at 324 nm we can observe: 1) three excited state bands immediately appearing; 2) a small rise of the signal at λ < 400 nm and at 500 nm together with the disappearing of signal at λ > 600 nm all in 100s of fs; 3) a recovery of GSB of **α** observed below 340 nm; a decrease of the overall signal in 10s of ps.

SVD-GF analysis, summarized in Figure S9B, confirms these results and quantifies the time components of these dynamics. According to this analysis, 5 components with time constants $\tau_{1}$~ 0.04 ps, $\tau_{2}$~ 0.13 ps, $\tau_{3}$~ 3 ps, $\tau_{4}$~ 70 ps, $\tau_{5}$ $\infty$ are necessary to take into the account all the relevant dynamics. At early times there is a pulse-limited decay ($\tau_{1}$) of the ESA which is initially populated. This is followed by another ultrafast dynamic ($\tau_{2}$~ 0.13 ps) mainly describing a decay of the ESA at λ > 600 nm and a small rise at 340 nm, λ<400 nm and at 500 nm. To interpret the sub-ps dynamics we can refer to the **P** monomer photocycle summarized in the introduction and reported in ref. ^[5]^. Indeed, first we do not expect a dramatic difference between the potential energy surfaces of **P** and **α**; second the sub-ps dynamics in **α** are very similar to the dynamics of $\tau_{{IC}^{'}}$and $\tau_{{IC}^{''}}$ in **P** monomers, where we also found a simultaneous decay (at λ > 600 nm) and rise (at 340 nm and λ <400 nm).Accordingly, $\tau_{1}$ describes the departure from Franck-Condon region, whereas $\tau_{2}$ the *S_B_*→*S_A_* IC processes. The main difference between **α** and **P** photocycles is that in the former we found only one time constant for the IC. Likely the second IC process in **α** monomers happens faster than in **P** and $\tau_{{IC}^{''}}$ cannot be distinguished within our time resolution from $\tau_{{IC}^{'}}$. $\tau_{3}$dynamic shows a small shift of all the three bands, on a time scale typical of vibrational energy transfer (VET) and internal vibration relaxation (IVR), pointing to cooling. These dynamics are followed by a decay of the signal in 70 ps ($\tau_{4}$), compatible with a rotational diffusion process. Eventually the signal ends up with a long-lived signal, whose time constant $\tau_{5}$ is set to infinity being much longer that the spanned delay interval.

## Multichromophoric systems with alkynyl phenanthrenes without acceptor (MCS1-A)

Moving to the MCS1-A, the stacking causes a spectral broadening and less structured bands (Figure S9) with respect to the TA spectra of the monomer (Figure S8). We also observe a pulse limited decay of the red part of ESA band at λ > 600 nm, which resembles what found in MCS1-C ($\tau_{1}$).

As for **α**, 5 characteristic time were enough to describe the spectral evolution (Figure S9B). The first DAS confirms the pulse-limited decay of ESA populated at time zero and it is followed by a fast decay of a broad band in 240 fs ($\tau_{2}$).

| 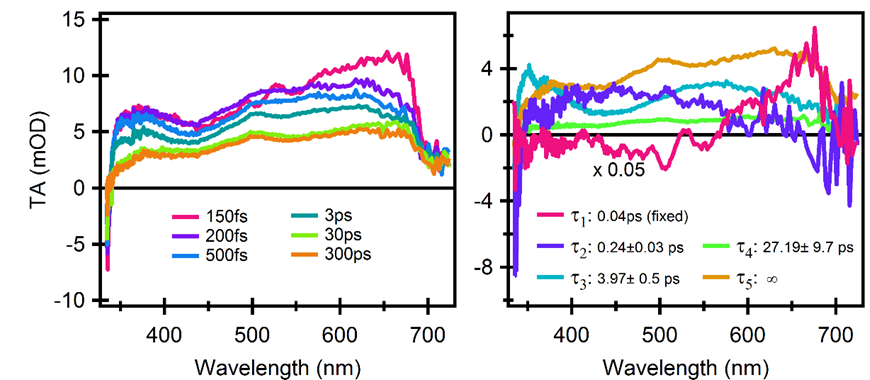  Figure S9. Ultrafast TA spectroscopy on MCS1-A. (A) A representative selection of TA spectra. (B) Decay associated spectra and respective decay time constants from the SVD-GF analysis. |
| --- |

Similar to MCS1-C ($\tau_{{IC}^{'}}$), this band is very broad and unstructured in agreement with ESAs due to delocalized electronic states generated by the stacking of several **α**s. It also contains an important negative signal below 340 nm, which speaks for a decay of the GSB of stacked **α**s. These dynamics are substantially equivalent to the one observed for the MCS1‑C and we can safely state that *DAS_2_* describes the decay of a broadband delocalized state, populated immediately after excitation, towards a localized lower excited state. Analogously to **α** monomers, we cannot distinguish between $\tau_{{IC}^{'}}$ and $\tau_{{IC}^{''}}$. Also the ps dynamics, $\tau_{3}$ and $\tau_{4}$, are very similar to what we observed in MSC1-C monomers and can be straight forwardly assigned to small cooling and relaxation of $S_{B}$ ($\tau_{3}$) followed by structural reorganization of DNA-hosted MCS array ($\tau_{4}$). Noteworthy, as for the case of the MCS1-C and with similar arguments, we can exclude that the 27 ps component is due to rotational diffusion but it describes a structural reorganization of DNA-hosted MCS array. Eventually we identified a long-lasting component ($\tau_{5}$) describing the recovery of ground state.

# FRET calculations

This analysis of the MCS2‑A reported in the main text confirmed the presence of different ET channels: one channel funnels part of excitation energy to **Y** immediately, which can only be via coherent ET mediated by delocalized state, whereas other channels transfer the energy to **Y** after it is localized on a $S_{A}$ state of one **α**. Because of the so packed geometry and the spectral overlap of **α** emission and **Y** absorption spectra, the most probable origin for these relatively slow ET channels is Förster Resonance Energy Transfer (FRET). The analysis in this section proves that a FRET mechanism is at the base of the slow ET channels.

Herein we report the analysis of the time constants describing the rise of the **Y** population in the MCS2-A in terms of FRET from each phenanthrene **α** to the pyrene **Y**. According to this ET mechanism the donor-acceptor (D-A) FRET rate, *k_FRET_*, is a function of the D-A distance, *R*, the mutual orientation and the natural depopulation rate of the donor photo-excited state, *k_D_*:^[18,19]^

| $\frac{k_{FRET}}{k_{FRET}+k_{D}}= \frac{1}{1+\left( \frac{R}{R_{0}} \right)^{6}\Gamma^{2}}$ | eq. S3 |
| --- | --- |
| $\Gamma^{2}={[\vec{\mu}_{D}.\vec{\mu}_{A}-3(\vec{r}_{DA}.\vec{\mu}_{D})(\vec{r}_{DA}.\vec{\mu}_{A})]}^{2}$ | eq. S4 |

where *R_0_* is the so-called Förster distance, a parameter that quantifies the D-A coupling range. *k_D_* is the total depopulation rate of the donor photo-excited state without the acceptor, namely all the radiative and non-radiative relaxation channels except the FRET one. Γ^2^ is the orientation factor which reflects that the ultimate nature of FRET is a dipole-dipole interaction: $\vec{\mu}_{D(A)}$ is the unitary vector of the emission (absorption) transition dipole of the donor (acceptor) and $\vec{r}$_DA_ is the unitary acceptor-donor displacement, $\vec{R}$ = ${R\cdot\vec{r}}_{DA}$. Since the phenanthrene alone is emissive and the absorption cross-section is 35400 M^‑1^cm^‑1^, the natural lifetime of the donor τ*_D_* = *k_D_*^‑1^ must be few ns long. Accordingly FRET must be the dominant relaxation channel and the lifetime of the donor exited state τ can be written as:

| $\tau=\left( k_{FRET}+k_{D} \right)^{-1}\approx{k_{FRET}}^{-1}=\tau_{D}\left( \frac{R}{R_{0}} \right)^{6}\frac{1}{\Gamma^{2}}$ | eq. S5 |
| --- | --- |

In order to adapt these equations to the case of MCS2-A, we built the structural model in Figure S10 according to X-ray diffraction studies on similar systems,^[20]^ which estimate the distance and the transversal displacement between two adjacent chromophores to be 3.5 Å and 1 Å, respectively. Assuming for simplicity $\vec{\mu}_{A}$ and $\vec{\mu}_{D}$parallel, the orientation factor becomes:

| $\Gamma^{2}={[1-3{sin}^{2}(\vartheta_{DA})]}^{2}$ | eq. S6 |
| --- | --- |

where $\vartheta_{DA}$is the angle between $\vec{r}_{\mathrm{DA}}$ and the transition dipoles of the acceptor **Y** and of the *i*^th^ donor ***α****_i_* (see Figure S10 for its definition), with index *i*=1 to 4 labelling from the closest to the furthest **α**. The geometrical parameters of the model are reported in Table S2 where we extend the calculations to *i*=5 and 6 for the sake of completeness. To compare the experimental value $\tau_{i}^{ET}$ with the FRET model it is more convenient to correct them by the respective orientation factor

| $\tau_{i}^{0}=\tau_{i}^{ET}\Gamma_{i}^{2}$ | eq. S7 |
| --- | --- |

and then to check if $\sqrt[6]{\tau_{i}^{0}}$ shows a liner dependence on R with zero intercept (see eq. S5):

| $\sqrt[6]{\tau_{i}^{0}}=\sqrt[6]{\tau_{i}^{ET}\Gamma^{2}}=\frac{\sqrt[6]{\tau_{D}}}{R_{0}}R_{i}$ | eq. S8 |
| --- | --- |

Alternatively, we can calculate $R_{0}$:

| $R_{0}=R_{i}\sqrt[6]{\frac{\tau_{D}}{\tau_{i}^{ET}\Gamma_{i}^{2}}}=R_{i}\sqrt[6]{\frac{\tau_{D}}{\tau_{i}^{0}}}$ | eq. S9 |
| --- | --- |

and verify how much each independent estimation is consistent with a constant value regardless the donor.

About *d_i_*, we set *d_i_*=0 Å and 1 Å for even and odd indexes, respectively, in agreement with the X-ray crystallographic data. The inter-chromophore distance *R_i_*, $\vartheta_{DA}$ and Γ^2^ are calculated as (*D^2^*+*d^2^*)^1/2^, tg^‑1^(^d^/_D_); and by means of eq. S5, respectively. These geometrical parameters are reported in tables S2. The experimental values $\tau_{i}^{ET}$, the ones corrected by Γ^2^ (eq. S7) and the Förster distance, $R_{0}$ (eq. S9) are also reported in Table S2. To calculate $R_{0}$, a typical value of 10 ns for $\tau_{D}$ was assumed based on the measured luminescence lifetime for phenanthrene monomers reported in ^[21]^. A different value of $\tau_{D}$would change the estimation of $R_{0}$ but not the overall interpretation. For the sake of completeness, we also report in Table S2 $R_{0}^{'}$, the estimation for *R_0_* without the correction by the orientation factor (or as if *d_i_* and therefore $\vartheta_{{DA}_{i}}$ were zero for all the **α**_i_).

Figure S11 shows these values of $\tau_{i}^{ET}$, $R_{0}^{'}$ (red rounds) and $\tau_{i}^{0}$ and *R_0_* (green diamonds) as a function of the donor‑acceptor distance. Except for the first point, the excellent agreement between $\tau_{i}^{ET}$ and $\tau_{i}^{0}$ with a R^6^ power law and the fact that estimations of *R_0_* are the same regardless the donor position, definitely confirm that the ET dynamics experimentally measured fit to a FRET model with a Förster distance of (26.9 ± 0.9) Å.

| 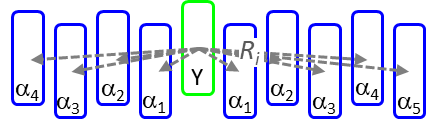  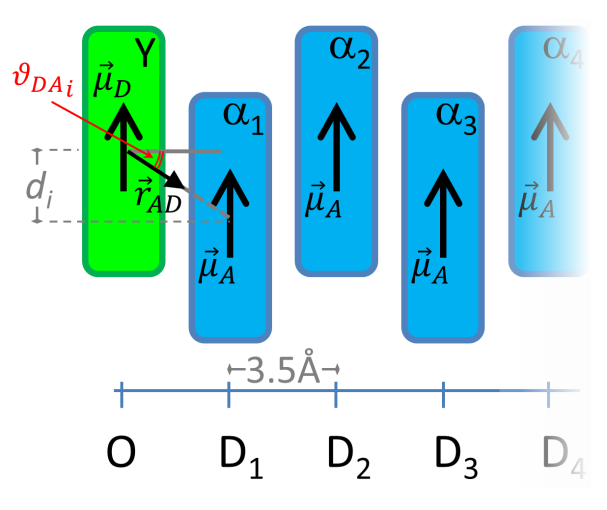  Figure S10. Structural model used to calculate the FRET rate for each donor. The relevant geometrical parameters and calculated outcomes are reported in Table S2. The complete stack is sketched in the top panel, while geometrical parameters and relevant vectors are defined in the bottom panel. For visualization purposes, only the first three donors **α** close to the acceptor **Y** (blue and green blocks, respectively) are shown. |
| --- |

The agreement of the last three points is excellent, whereas the first shows a discrepancy that is only partially corrected by the geometrical factor. However, if we allow the pyrene to displace vertically by 0.84 Å also this point perfectly matches with a 6^th^ power law (Table S3 and blue squares in Figure S11). It is indeed reasonable to expect for the pyrene some deviation from the ordered staking, since it is different from the other chromophores, namely phenanthrenes. Within the proposed structural model, the possible distortions are a change in the longitudinal or transversal inter-chromophore distance. In the former case, it would be necessary to increase the longitudinal distance between the **Y** and the closest **α**s from 3.5 Å to 5.25 Å, which corresponds to $R_{1}$= 5.34 Å, $\tau_{1}^{0}$= 0.60 ps and $R_{o}$=27.0 Å. In the latter case it is enough to set *d_1_*=1.84 Å to obtain parameters ($R_{1}$ = 3.95 Å, $\tau_{1}^{0}$= 0.09 ps and $R_{o}$ = 27.3 Å, see table S3) in excellent agreement with the R^6^ power law (see Figure S11) and $R_{0}$= 27 Å. We consider the latter the only possible since it requires only a deviation 0.3 Å instead of 1.6 Å with respect to the crystallographic data (3.95 Å and 5.25 Å, respectively, against 3.64 Å); it mainly concerns only the first donor with minor effects on the distance between the acceptor and the other donors and a small parallel dislocation is energetically more favorable than a distance change along the staking axis. Probably the real geometry implies also a small change in the relative orientations between **Y** and ***α****_1_* but these simple calculations show that a relative shift is enough.

For the sake of completeness, the data plotted in Figure 4 are the $\tau_{i}^{ET}$and the $\tau_{i}^{0}$ from Table S3 and plotted in Figure S11 with the same symbols (red rounds and blue squares, respectively).

The comparison of $\tau_{i}^{ET}$ and $\tau_{i}^{0}$ points out that taking into account the spatial arrangement of the chromophores is crucial mainly for the 1^st^ **α**, since it has the less favorable angle $(\vartheta_{{DA}_{1}}$) and definitively bigger than $\vartheta_{{DA}_{2}}$ and $\vartheta_{{DA}_{3}}$ while, going further from the **Y**, the transversal displacement becomes less and less critical.

Table S2. The geometrical parameters used for the structural model depicted in Figure S10 and used for the FRET calculation: *D_i_* and *d_i_*, are the longitudinal and transversal displacements of the *i*^th^ donor with respect to the acceptor, *R_i_* is the inter-chromophore distance calculated from *D_i_* and *d_i_*, $\vartheta_{{DA}_{i}}$ is the angle between the transition dipoles of the acceptor and *i^th^* donor. $\tau_{i}$ are the experimental FRET time constants from the analysis of MSC2-A; $\tau_{i}^{0}$are equal to $\tau_{i}$ corrected by the orientation factor (eq. S7). $R_{0}$ is the Förster distance calculated according to eq. S9, while $R_{0}^{'}$ is calculated without considering the orientation factor (namely still from eq. S9 where $\tau_{i}^{0}$ is replaced by $\tau_{i}$ or equivalently *d_i_*=0 for any index). Reported errors are standard deviations. Data in the last two rows are extrapolated points calculated imposing *R_0_*=27 Å. ^(1)^ Calculated; ^(2)^ fixed value.

| Geometrical parameters | | | | Energy transfer (acceptor rise) time constants ($\tau$) | | Förster distance ($R_{0}$) | |
| --- | --- | --- | --- | --- | --- | --- | --- |
| *D_i_*  (Å) | *d_i_*  (Å) | $R_{i}$  (Å) | $\vartheta_{{DA}_{i}}$  (deg) | $\tau_{i}^{ET}$  (ps) | $\tau_{i}^{0}$  (ps) | $R_{0}$  (Å) | $R_{0}^{'}$  (Å) |
| 3.5 | 1 | 3.6 | 15.9° | 0.75±0.17 | 0.45±0.10 | 19.3±0.7 | 17.0±0.7 |
| 7 | 0 | 7 | 0 | 3.3±0.6 | 3.3±0.6 | 26.6±0.8 | 26.6±0.8 |
| 10.5 | 1 | 10.6 | 5.4° | 33±7 | 31±7 | 27.6±1.0 | 27.2±1.0 |
| 14 | 0 | 14 | 0 | 200±100 | 200±100 | 26.9±2.5 | 26.9±2.5 |
| 17.5 | 1 | 17.5 | 3.3 | 790^(1)^ | 775^(1)^ | 27^(2)^ |  |
| 21 | 0 | 21 | 0 | 2100^(1)^ | 2100^(1)^ | 27^(2)^ |  |

Table S3. As table S2 but assuming a positive vertical displacement of the acceptor by 0.84 Å. Accordingly, the only geometrical difference with respect to table S2 is the second column. The values in the other columns are calculated accordingly. ^(1)^ Calculated; ^(2)^ fixed value.

| Geometrical parameters | | | | Energy transfer (acceptor rise) time constants ($\tau$) | | Förster distance ($R_{0}$) |
| --- | --- | --- | --- | --- | --- | --- |
| *D_i_*  (Å) | *d_i_*  (Å) | $R_{i}$  (Å) | $\vartheta_{{DA}_{i}}$  (deg) | $\tau_{i}^{ET}$  (ps) | $\tau_{i}^{0}$  (ps) | $R_{0}$  (Å) |
| 3.5 | 1.84 | 4.0 | 27.7° | 0.75±0.17 | 0.09±0.02 | 27.3±1.1 |
| 7 | 0.84 | 7.1 | 6.8° | 3.3±0.6 | 3,0±0.5 | 27.2±0.8 |
| 10.5 | 1.84 | 10.7 | 9.9° | 33±7 | 27±6 | 28.5±1.0 |
| 14 | 0.84 | 14.0 | 3.4° | 200±100 | 200±100 | 27,0±2.5 |
| 17.5 | 1.84 | 17.6 | 6.0° | 790^(1)^ | 740^(1)^ | 27.2^(2)^ |
| 21 | 0.84 | 21.0 | 2.3° | 2100^(1)^ | 2080^(1)^ | 27.2^(2)^ |

| 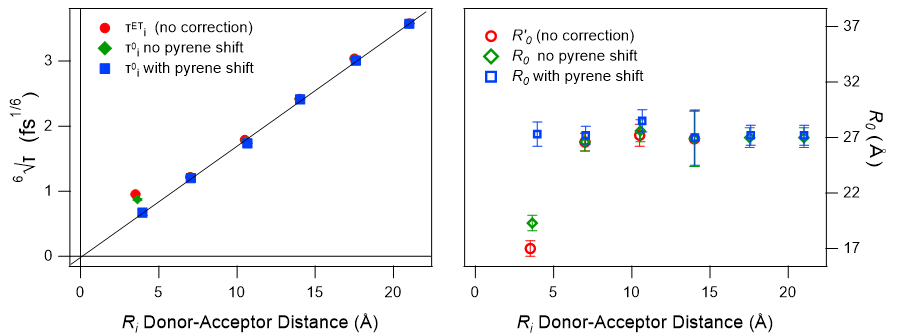  Figure S11. Comparison of the (left panel, solid symbols) energy transfer time constants and (right panel, empty symbols) Förster distances for the three geometrical configurations and reported in Table S2 and Table S3: (rounds) no vertical dislocation between odd and even chromophores (*d_i_*=0 for any *i*); (diamonds) odd and even chromophores are vertically displaced by 1 Å; (squares) odd and even chromophores are vertically displaced by 1 Å and the pyrene is shifted outwards by 0.89 Å. |
| --- |

# Energetic levels and exciton delocalization for phenanthrene pentamer and the pyrene‑phenanthrene dimer in parallel, helix and antiparallel conformations.

As discussed in the main text we resorted to a simple model system consisting of five α-stacked phenanthrene units, to rationalize the observed spectroscopic evolution, and clarify the involved electronic states and transitions. Coherently with the results of quantum chemistry we will use an adiabatic notation for the excited states (S_0_, S_1_, S_2_, …) based on the energy order. This can be also related to the S_A_ and S_B_ diabatic notation used previously and based, instead, on the nature of the states. To minimize the computational effort and avoid any spurious effects due to the change in the conformational flexibility, while concentrating instead on the inherent electronic properties of the MCSs, we discarded the peripheral substituents keeping only the aromatic cores.

All the calculations were performed in vacuo, since the inhomogeneous experimental environment could not be reproduced by implicit solvation models, and the use of hybrid quantum/classical methods coupled to a dynamic sampling of the conformational space would induce effects due to thermal disorder. Even if some solvatochromic shift can be expected the energy difference between local and delocalized states is such that inversion may be reasonably discarded, confirming the validity of our conclusions. In this respect, the chosen functional is known to be able to correctly reproduce the excited states manifold of organic chromophores, and it also include dispersion correction allowing to describe π-stacked arrangements. Furthermore, the same functional was already used to describe the excitation spectra, and the photophysical evolution of phenantrene also through surface hopping non-adiabatic dynamics.^[5]^

We manually built and compared three limit conformations composed of a parallel and antiparallel arrangement, as well as a helical structure that can be thought as intermediate between the formers (Figure S12). The excited states manifold as well as the topological analysis of the excited state electronic density reorganization for the three configurations are compared in Figure S12 (see Figure 5 for the helical conformation only). The excitation energy with respect to the ground state is reported in eV, the exciton delocalization is pictorially shown via the size of the box, and the oscillator strength via the corresponding color code. Note that the position of the exciton center of charge, i.e. its localization relative to the phenanthrene units is also represented by the position of its center along the x-axis.

While the antiparallel and helical arrangement provide a similar topology of the potential energy surface, the parallel conformation presents a much more peculiar behaviour. Indeed, in the case of the latter we can identify a quite bright and relatively local state centred on the central phenanthrene units and positioned at around 4.35 eV. After the initial absorption, this state could relax by internal conversion to a manifold of quasi-degenerate, rather delocalized states that would span over almost the entire column. It is important to remind that we did not observe a full delocalization due to the border effects inherent to a finite system, and also due to the specific algorithm used for the topological density analysis which is based on projection of density and hence tends to slightly underestimate the number of delocalized units. In the other two arrangements instead the oscillator strength of the absorbing state is lower, however the state is still quite localized, a first manifold of delocalized states extended over the full MCS can then be observed. However, this metastable state can evolve through the relaxation to the S_1_ state that is better localized and basically spans only one phenanthrene unit. The differences between the antiparallel and the helical state are mainly related to a more localized nature of the S_1_ state for the latter, also accompanied by a slight overstabilization of the lowest excited state not exceeding 0.1ev. The same stabilization in the helical arrangement compared to the antiparallel is also evidenced for the absorbing state, however its limited magnitude can still be easily overcome by thermal motion. These observations are also confirmed by the attachment and detachment densities, reported in Figure 5 for the principal excited states of the helical conformation.

|   Figure S12. A) Energetic level diagram and visualization of exciton delocalization for the stacked phenanthrene pentamer in parallel (B), helix (C), and antiparallel (D) conformation. |
| --- |

Furthermore, this scenario, while excluding the parallel conformation, also provides a coherent rationalization of the observed spectral features. Indeed, the energy level and the topology of the principal excited states suggest that after photon absorption by a local, i.e. monomer-like state, the system will evolve into a short-lived delocalized excitonic state that is instrumental to allow long-range coherent energy transfer, before relaxing to an emissive local and monomer-like state. Obviously, the exact unit on which the emissive S_1_ state will localize depend on the differences induced by thermal disorder that could change the exciton localization. However, as already discussed in the main text, it is important to underline that we have optimized the multichromophoric aggregate from and ideal symmetric situation, i.e. artificially favouring delocalization, hence the existence of localized S_1_ state cannot be seen as a computational artefact.

More importantly, the deexcitation cycle involving absorption to a localized state, delocalization, and subsequent relocalization to an emissive state, is observed both for antiparallel and helical conformation, suggesting the robustness of these feature that should persist even in presence of thermal agitation. Indeed, the parallel conformation is the only one which presents a rather different behaviour, and a smaller amount of delocalization. However, as previously said this conformation is most unlikely to be the present in the experimental system due to the constraints imposed by the DNA scaffold. This different behaviour can be seen as a confirmation of the importance of the presence of alternating arrangement of phenanthrene to promote delocalization.

| 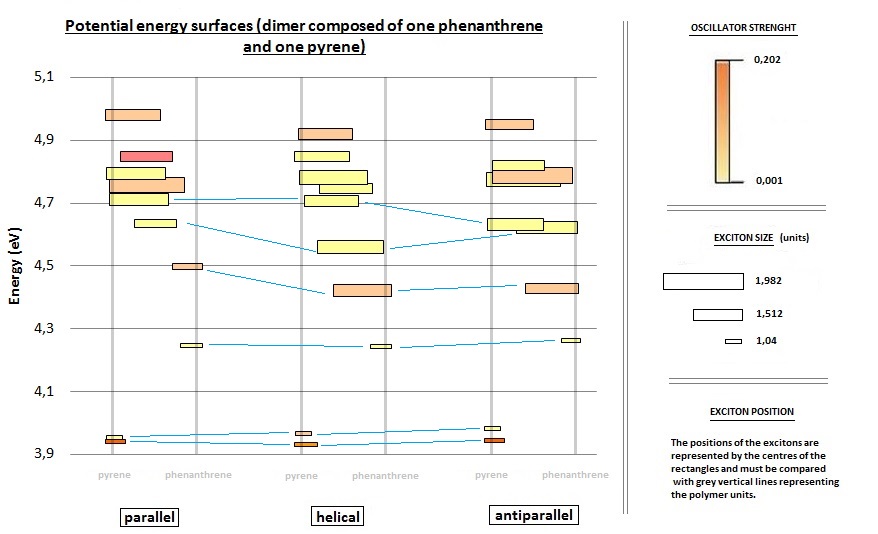  Figure S13. Energy diagram and characterization of the excitons for a pyrene/phenanthrene monomer. |
| --- |

Finally, to get more insight on the phenanthrene/pyrene energy transfer phenomena we also modelled the excited state landscapes of a stacked dimer in parallel, helical, and antiparallel orientations. The most relevant case of the helicoidal arrangement is also reported in Figure 6, while all the three cases are compared in Figure S13. As a general comment the overall outcome does not depend dramatically on the specific orientation, as is somehow expected considered the more symmetric geometry of the pyrene (Scheme S1)

Accordingly, the discussion reported in the main text about the helical configuration applies also to the parallel and the antiparallel arrangements.

# Comment on the coherent nature of the energy transfer mechanism

We should observe that being an energy transfer ultrafast does not mean to be a coherent process, because it could be, in principle still and ultrafast uncoherent (hopping) process. The main distinction is that the former is *fully* mediated by delocalized (exitonic) states or superposition of states, whereas the latter is a population hopping from electronic states localized on each molecule. For the sake of completeness, we could have an intermediate situation as in natural light-harvesting complexes’ systems, where the uncoherent hopping occurs between excitons localized over few molecular units. For this reason, first we proved with the computational study reported in the main text that such delocalized excitonic states exist and can sustain the coherent ET process.

However, this is still not enough because the interchromophore coupling originating the excitonic states, must be strong enough against the thermal fluctuations allowing the occurrence of the coherent mechanism. We can indirectly estimate the order of magnitude of the strength of the coupling from the rate of the ET process. Indeed, the fact that we do not observe any rise of the acceptor points to the fact the, even from the further donor, the energy is fully transferred within our time resolution (40 fs). The further donor is 5 unit apart which would imply a hopping rate between two P units of > (8 fs)^‑1^. Considering the indirect evidence from steady state measurements, which report an ET efficiency over 8 (Angew. Chem. Int. Ed. 2012, 51, 916 –919; Bioconjugate Chem **2012**, *23*, 2105-2113) or even 9 (Org. Biomol. Chem.,2020, 18,6818–6822) units, this value is very likely close to 1 fs^‑1^. If the process would be uncoherent, as the case of hopping, we are allowed to use the Fermi’s golden rule to calculate the strength of the coupling:

| $\sqrt{\frac{\hbar}{2\pi}\Gamma}=0.32 eV$ | eq. S10, |
| --- | --- |

with Γ = 1 fs^‑1^. In this derivation we omitted the density of state of the final state, which would originate from the coupling with the vibrational modes. We are allowed to set it to unity because the origin of the exitonic coupling is a π‑π staking, which act on the electronic coordinates only. In addition, on the time scale of 1 fs no nucleus can significantly change its momentum. For these two reasons, we can consider the energy transfer as a process between two degenerate states with the same vibrational configuration. A value of 0.32 eV is definitively higher than *k_B_T* at room temperature (25 meV) and it is not compatible with the condition at the base of hopping, namely interchromophore coupling weaker than thermal fluctuations.

Therefore, the fact that the acceptor is immediately populated, and the existence of robust excitonic states are strongly supportive of the occurrence of a coherent superposition of states.

# References:

[1] S. M. Langenegger, R. Häner, *Helv. Chim. Acta* **2002**, *85*, 3414–3421.

[2] S. M. Langenegger, R. Häner, *Chem. Commun. (Camb).* **2004**, 2792–2793.

[3] H. Bittermann, D. Siegemund, V. L. Malinovskii, R. Häner, *J. Am. Chem. Soc.* **2008**, *130*, 15285–15287.

[4] C. B. Winiger, S. Li, G. R. Kumar, S. M. Langenegger, R. Häner, *Angew. Chem., Int. Ed.* **2014**, *53*, 13609–13613.

[5] M. Nazari, C. D. Bösch, A. Rondi, A. Francés-Monerris, M. Marazzi, E. Lognon, M. Gazzetto, S. M. Langenegger, R. Häner, T. Feurer, A. Monari, A. Cannizzo, *Phys. Chem. Chem. Phys.* **2019**, *21*.

[6] M. Nazari Haghighi Pashaki, N. Mosimann-schönbächler, A. Riede, M. Gazzetto, A. Rondi, A. Cannizzo, *J. Phys. Photonics* **2021**, *3*, 34014.

[7] A. Cannizzo, A. M. Blanco-rodríguez, A. El Nahhas, J. Šebera, S. Záliš, A. Vlček Jr., M. Chergui, *J. Am. Chem. Soc.* **2008**, *130*, 8967–8974.

[8] *Gaussian 09 Revis. D.01* **2009**, 2–3.

[9] J. Da Chai, M. Head-Gordon, *Phys. Chem. Chem. Phys.* **2008**, *10*, 6615–6620.

[10] T. Yanai, D. P. Tew, N. C. Handy, *Chem. Phys. Lett.* **2004**, *393*, 51–57.

[11] F. Plasser, *J. Chem. Phys.* **2020**, *152*, DOI 10.1063/1.5143076.

[12] N. M. O’Boyle, A. L. Tenderholt, K. M. Langner, *J. Comput. Chem.* **2008**, *29*, 839–845.

[13] F. Garo, R. Häner, *Angew. Chemie - Int. Ed.* **2012**, *51*, 916–919.

[14] F. Garo, R. Häner, *Bioconjug. Chem.* **2012**, *23*, 2105–2113.

[15] C. B. Winiger, S. M. Langenegger, O. Khorev, R. Häner, *Beilstein J. Org. Chem.* **2014**, *10*, 1589–1595.

[16] N. Bürki, E. Grossenbacher, A. Cannizzo, T. Feurer, S. M. Langenegger, R. Häner, *Org. Biomol. Chem.* **2020**, *18*, 6818–6822.

[17] C. D. Bösch, E. Abay, S. M. Langenegger, M. Nazari, A. Cannizzo, T. Feurer, R. Häner, *Helv. Chim. Acta* **2019**, *102*, e1900148.

[18] J. R. Lakowicz, *Principles of Fluorescence Spectroscopy*, Springer US, **2006**.

[19] T. Mirkovic, E. E. Ostroumov, J. M. Anna, R. Van Grondelle, Govindjee, G. D. Scholes, *Chem. Rev.* **2017**, *117*, 249–293.

[20] M. Probst, W. Aeschimann, T. T. H. Chau, S. M. Langenegger, A. Stocker, R. Häner, *Nucleic Acids Res.* **2016**, *44*, 7079–7089.

[21] B. Nickel, *Chem. Phys.* **1986**, *110*, 131–143.

[22] C. B. Winiger, S. Li, G. R. Kumar, S. M. Langenegger, R. Häner, *Angew. Chem., Int. Ed.* **2014**, *53*, 13609–13613.
